# Systematic modelling of the development of laminar projection origins in the cerebral cortex: Interactions of spatio-temporal patterns of neurogenesis and cellular heterogeneity

**DOI:** 10.1371/journal.pcbi.1007991

**Published:** 2020-10-13

**Authors:** Sarah F. Beul, Claus C. Hilgetag

**Affiliations:** 1 Institute of Computational Neuroscience, University Medical Center Hamburg-Eppendorf, Hamburg, Germany; 2 Neural Systems Laboratory, Department of Health Sciences, Boston University, Boston, Massachusetts, United States of America; Ghent University, BELGIUM

## Abstract

The architectonic type principle conceptualizes structural connections between brain areas in terms of the relative architectonic differentiation of connected areas. It has previously been shown that spatio-temporal interactions between the time and place of neurogenesis could underlie multiple features of empirical mammalian connectomes, such as projection existence and the distribution of projection strengths. However, so far no mechanistic explanation for the emergence of typically observed laminar patterns of projection origins and terminations has been tested. Here, we expand an *in silico* model of the developing cortical sheet to explore which factors could potentially constrain the development of laminar projection patterns. We show that manipulations which rely solely on spatio-temporal interactions, namely the relative density of laminar compartments, a delay in the neurogenesis of infragranular layers relative to layer 1, and a delay in the neurogenesis of supragranular layers relative to infragranular layers, do not result in the striking correlation between supragranular contribution to projections and the relative differentiation of areas that is typically observed in the mammalian cortex. In contrast, we find that if we introduce systematic variation in cell-intrinsic properties, coupling them with architectonic differentiation, the resulting laminar projection patterns closely mirror the empirically observed patterns. We also find that the spatio-temporal interactions posited to occur during neurogenesis are necessary for the formation of the characteristic laminar patterns. Hence, our results indicate that the specification of the laminar patterns of projection origins may result from systematic variation in a number of cell-intrinsic properties, superimposed on the previously identified spatio-temporal interactions which are sufficient for the emergence of the architectonic type principle on the level of inter-areal connectivity *in silico*.

## Author summary

The organization of structural connections in the brain is crucial for determining brain function. One feature of cortico-cortical connections is the distribution of their origins and terminations across cortical layers, which has been reported to exhibit a striking correlation with the architectonic differentiation of the connected cortical areas. How these laminar patterns emerge during development remains an open question. Here, we explored possible scenarios using systematic simulation experiments. We built on prior simulation experiments, in which we found that realistic patterns of connection existence may arise from spatio-temporal interactions in a homogeneous cortical sheet. We extended the previous *in silico* model of the developing cortical sheet by adding laminar compartments, to probe the development of laminar patterns of connection origins. We found that we could not easily generate characteristic laminar origin patterns by modifying the spatio-temporal growth trajectory of the homogeneous developing cortical sheet (i.e. changing when and where otherwise identical neurons were generated). However, when we added cellular heterogeneity, by modifying a property of axon growth across the simulated cortical sheet, the simulation was able to generate laminar patterns of connection origins that exhibited a positive correlation with the difference in architectonic differentiation between connected areas, similar to the relationship that can be observed in the mammalian cortex.

## Introduction

Structural connections between cortical areas are the substrate for information processing in the brain. Their intricate organization still poses many questions, both regarding the fully developed adult state and the developmental processes shaping it. One comprehensive framework that captures many aspects of the organization of structural connectivity in the mammalian brain is the architectonic type principle [1–4]. It represents connections in terms of the relative architectonic differentiation between brain areas and has been shown to account well for multiple features of cortico-cortical projections across the entire cortex of different mammalian species [1,5–17]. Architectonic differentiation refers to the overall appearance of cortical areas with respect to a number of structural properties, such as their neuron density, the number of identifiable cortical layers, the density of myelin, and different receptor markers and specialized inhibitory neurons [7,18–21]. Differentiation ranges across a spectrum from eulaminate areas with a remarkable clarity of lamination and dense packing of neurons, such as striate cortex, to agranular areas that lack an inner granular layer (layer 4) and have few identifiable sublayers as well as low neuron density. Between these two extremes, there are eulaminate areas with a less clear-cut lamination as well as dysgranular areas with intermediate neuron density, a dissolving inner granular layer and fewer identifiable sublayers. The most intricate property of structural connections that is well captured by the architectonic type principle concerns the distributions of projection neurons’ somata across cortical layers. These laminar patterns of projection origins have been shown to vary gradually as the difference in architectonic differentiation between the two connected areas changes [1–4]. Specifically, a positive correlation has been observed, such that the contribution to a projection from the supragranular layers becomes stronger, the more differentiated the source area is compared to the target area. This meant that projections from areas of weaker differentiation are formed increasingly from infragranular layers as they target areas of increasingly stronger differentiation, while projections from areas of stronger differentiation are formed increasingly from the supragranular layers as they target areas of weaker differentiation.

It has been suggested that the architectonic type principle could emerge from spatio-temporal interactions in the developing brain [1,2,7,15], where correlations of time of origin with both distance between cortical areas (and thus their probability to connect to each other) and with the architectonic differentiation of cortical areas (where areas that are formed later are of stronger differentiation) would interact to result in the empirically observed correlations of connectivity with architectonic differentiation. We previously employed simulation experiments to show that such simple interactions could indeed result in patterns of cortico-cortical connectivity that resemble connectivity in the mammalian brain with respect to the existence [22] and strength [23] of connections. However, it is still unclear how the characteristic laminar patterns of projection origins arise that originally prompted the formulation of the architectonic type principle [1].

### Laminar patterns regulate information processing

The specific laminar composition of connections is crucial to their function, given that neurons in the different layers, through their differing morphology, are endowed with distinct processing capabilities. In fact, lamination itself may only be relevant to the extent that it reflects the arrangement of particular types of brain cells [24]. It has been shown that oscillations of particular frequencies dominate in different cortical layers [25–28]. Since these oscillations are associated with communication in specific directions (forward/backward) [28–31], they are likely related to the laminar patterns of cortico-cortical connections [28]. Moreover, oscillations across different frequency bands are a crucial feature in theories of brain function such as predictive coding [32], underlie executive processes such as working memory regulation (reviewed in [33]) and have been identified to be causal for self-reflective awareness in humans [34]. The laminar specifics of cortico-cortical connections therefore have implications for a wide spectrum of functions, and certain types of connections are crucial for processes extending to cognition and conscious perception in humans (reviewed in [35]).

Hence, integrating the characteristics of cortico-cortical connectivity with intrinsic circuitry in source and target areas is important for understanding experimental results. This integration is, however, also profoundly useful in deriving powerful models of cortical function. For example, validated regularities can be harnessed to infer missing data points in empirical data sets and build better performing models than possible with the incomplete data alone. This approach has, for example, been gainfully employed in the construction of computational models of cortical network function [36,37].

### An *in silico* model to probe the emergence of laminar patterns of projection origins

Observing the developmental events that shape cortico-cortical connectivity during the course of ontogenesis in sufficient detail to answer the question of how laminar patterns emerge remains challenging at best. Therefore, we extended our *in silico* model of cortical development to explore some features that could potentially be relevant for the formation of laminar projection patterns.

We have shown previously, that the architectonic type principle is already applicable to laminar patterns of projection origins at early stages of development [38]. Therefore, we limited our exploration to features that would affect patterns of connectivity early in development and disregarded later occurring processes such as regressive events and activity-dependent remodelling of projections. This is consistent with observations showing that the mouse brain can form in a typical manner, including initial connectivity, independent of synaptic transmission [39].

**Fig 1:**
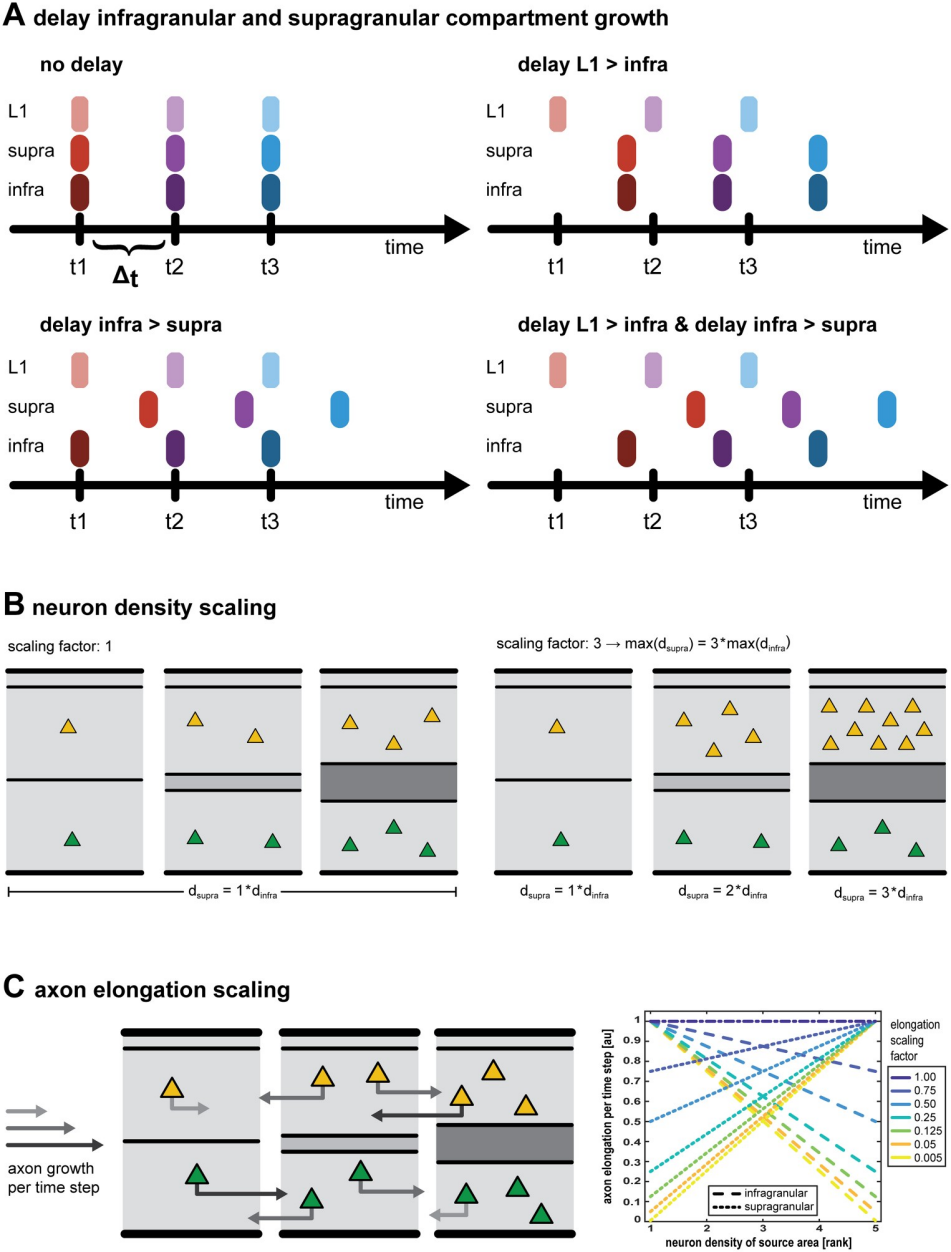
Features of the *in silico* model. (A) Delay in the growth of laminar compartments. Without a delay in compartment growth (*no delay*), all laminar compartments of a given area grow at one single time point. After the growth interval, Δ_t_, the next area appears. If growth of the infragranular compartment is delayed relative to layer 1 (*delay L1 > infra*), the infragranular compartment grows a fraction of the growth interval after layer 1, while the supragranular compartment appears simultaneously with the infragranular compartment. If growth of the supragranular compartment is delayed (*delay infra > supra*), it grows a fraction of the growth intervals after layer 1 and the infragranular compartment, both of which appear simultaneously. If both compartments are delayed (*delay L1 > infra & delay infra > supra*), layer 1 appears first, followed by the infragranular compartment and finally the supragranular compartment. (B) Scaling in the neuron density of the supragranular compartment. With a scaling factor for supragranular density larger than 1, the ratio of supragranular neuron density to infragranular neuron density becomes larger as infragranular neuron density increases across areas. Additionally, as the scaling factor becomes larger, the divergence between low-density and high-density areas in their ratio of supragranular to infragranular neuron density increases. (C) Scaling in axon elongation. We modified how much longer axons became at each time step according to both the laminar compartments of the neuron somata and the architectonic differentiation of the area the neuron somata were positioned in. Axon elongation was gradually adjusted to shrink to a minimum value (light grey arrow), with the ratio of minimum elongation to baseline elongation given by the elongation scaling factor (see color scale). As the scaling factor became smaller, the divergence in elongation values became larger. We implemented two opposing gradients: elongation values in the infragranular compartment (dashed lines) became shorter with increasing source area neuron density, while elongation values in the supragranular compartment (dotted lines) became longer with increasing source area neuron density. At a scaling factor of 1, all neurons, regardless of laminar compartment or source area neuron density, shared the same elongation value (appears as dash-dotted line).

In the simulation experiments reported here, we tested the effect of adding four features to the *in silico* model (Fig 1): a delay in the time point of neurogenesis of the infragranular compartment relative to layer 1, a delay in the time point of neurogenesis of the supragranular compartment relative to the infragranular compartment, a scaling of the neuron density in the supragranular compartment, and a scaling of the elongation of neurons’ axons. The first three of these features modified spatio-temporal patterns of neurogenesis, while the fourth feature affected properties of individual neurons.

The two delays in the growth of the laminar compartment straightforwardly mirror the radial gradient in neurogenesis that can be observed for cortical neurons [40–43] by assigning neurons to birth cohorts according to laminar compartments. With the exception of layer 1 neurons (which are formed first), neurons that are born later come to populate successively more superficial positions in the cortical sheet. Thus, the cortical sheet forms in an inside-out manner with infragranular layer neurons at a particular position of the cortical sheet born before neurons in the supragranular layers.

As architectonic differentiation becomes stronger and neuron density becomes higher, density increases especially in the supragranular layers of the mammalian cortex [44–46]. In the primate cortex, the comparatively higher supragranular neuron density is effected by an expansion of the subventricular zone relative to other species, as for example rodents, to the extent that it is split into distinct compartments of the inner and outer subventricular zone [47]. The cell cycle kinetics underlying the transition from progenitor cells to differentiated neurons have been described in detail [47–52] and explain a selective supragranular increase. As neurogenesis progresses across the cortical sheet, cell cycles lengthen and the proportion of progenitor cells that differentiate into neurons successively increases with each cell cycle. At the level of cortical areas, this results in a positive correlation between time of origin and neuron density [44,53]. In addition, since later cycles lengthen the most and yield neurons destined for the upper layers, as cycles become longer and overall neuron density increases, the effect is particularly pronounced in the supragranular layers [54,55]. We implemented this notable increase in relative supragranular neuron density by scaling the neuron density of the supragranular compartment to be relatively higher than infragranular compartment neuron density, and this difference to be larger the more differentiated an area was.

As architectonic differentiation becomes stronger, there are many changes beyond an increase in neuron density. For example, myelination, cellular markers of synaptic stability and plasticity, as well as neurotransmitter receptor complement change across the spectrum of architectonic differentiation [7,56–60], and properties of cell morphology such as soma cross section, dendritic tree size and dendritic spine count are correlated with neuron density [17]. As a final modification, we therefore probed which effects on laminar projection patterns could arise from changes to cell-intrinsic properties across the differentiation spectrum. We chose to manipulate axon elongation, because two observations, detailed below, create a tentative link between gradual changes in architectonic differentiation and the laminar position of cells best equipped for maintaining longer projections. First, there is a striking shift in the laminar distribution of larger neurons across the cortex, which has been termed externopyramidization [61,62]. Depending on whether larger neurons are predominantly found in the infragranular or in the supragranular layers, areas can be classified as internopyramidal or externopyramidal, respectively. Thus, in internopyramidal areas the ratio of supragranular neuron size to infragranular neuron size is smaller than it is in externopyramidal areas. This ratio of neuron sizes changes gradually across the cortex and coincides with the degree of architectonic differentiation, such that supragranular neuron size tends to be relatively larger compared to infragranular neuron size in more differentiated areas (reviewed in [63]). Second, multiple lines of evidence suggest that neuron size is related to axon length (reviewed in [63]) (although this does not speak to the direction of the causality, that is, whether larger neurons maintain longer connections or whether the formation of longer connections induces neuron somata to become larger). For example, considerations of metabolic cost [64,65], attainable conduction velocities [66,67] and synaptic efficacy [68–71] suggest that larger neurons are particularly capable of maintaining projections across larger distances. Taken together, these observations suggest that in areas of different architectonic differentiation the neurons that are best suited for forming longer projections are situated in different layers, and thus, as hypothesized before [63,72], that externopyramidization might be associated with shifts in laminar patterns of projection origins. We therefore constructed our *in silico* manipulation of axon elongation to mirror the changes in relative neuron size observed across the spectrum of architectonic differentiation.

Here, we present results obtained from modifying an *in silico* model of cortical histogenesis, probing how the distribution of projection origins across laminar compartments was affected either by changes in the spatio-temporal patterns of neurogenesis or by gradual changes in cell-intrinsic properties. Our simulation experiments only replicated changes in laminar origin patterns which are observed empirically across the spectrum of architectonic differentiation when we modified cell-intrinsic properties, suggesting that factors beyond spatio-temporal interactions in the forming cortical sheet mediate the specification of laminar projection patterns.

## Results

**Fig 2:**
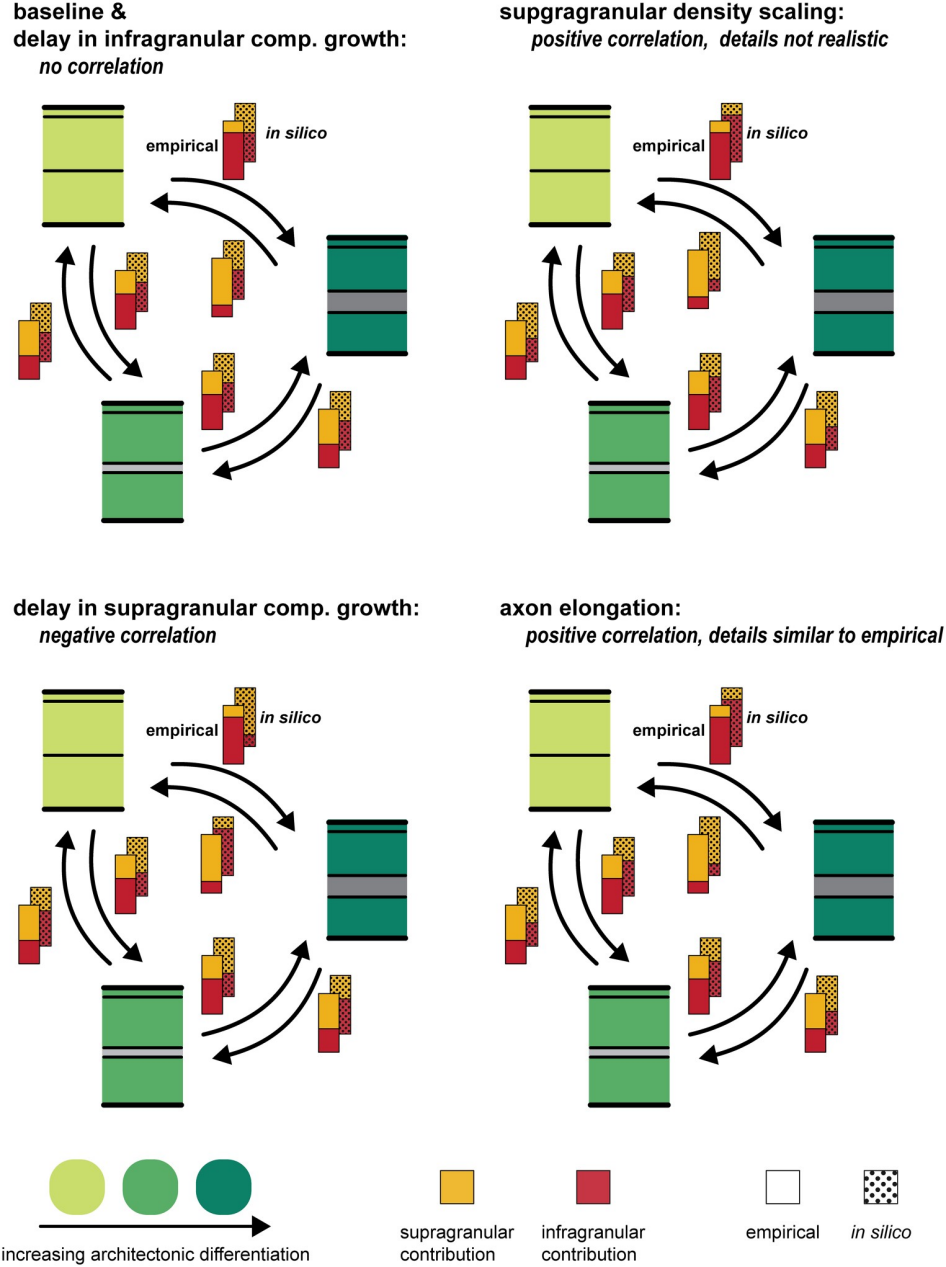
Overview of observed correlations between supragranular contribution and neuron density difference. Depending on which feature was implemented in the *in silico* model, the correlation between supragranular contribution and neuron density difference changed (stippled bars). Empirically, a positive correlation has been observed (solid bars): the layer from which a connection originates shifts depending on the differentiation of the source area compared to the target area. At the baseline setting, the *in silico* model did not produce such a correlation, Instead, all connections were formed at about equal proportions by neurons from both the infragranular and the supragranular compartment, and the differentiation of source area or target area did not have any influence. If the delay in infragranular compartment growth was implemented, the correlation was not affected (Section 2.1). A delay in supragranular compartment growth resulted in a negative correlation between supragranular contribution and neuron density difference (Section 2.2). Implementing the scaling of supragranular neuron density led to a positive correlation, which did not match the empirical laminar patterns in its details and was abolished by controlling for the ratio of supragranular neurons to total neurons (Section 2.3, Figs 4 and 5). The scaling of axon elongation, finally, resulted in a positive correlation that emerged from laminar patterns which were similar to the empirically observed laminar patterns (Section 2.4).

We systematically explored the effects of including three features affecting spatio-temporal patterns of neurogenesis and one feature affecting cell-intrinsic properties on the laminar patterns of projection origins in an *in silico* model of the developing cortical sheet. We give an overview of the results in Fig 2.

**Fig 3:**
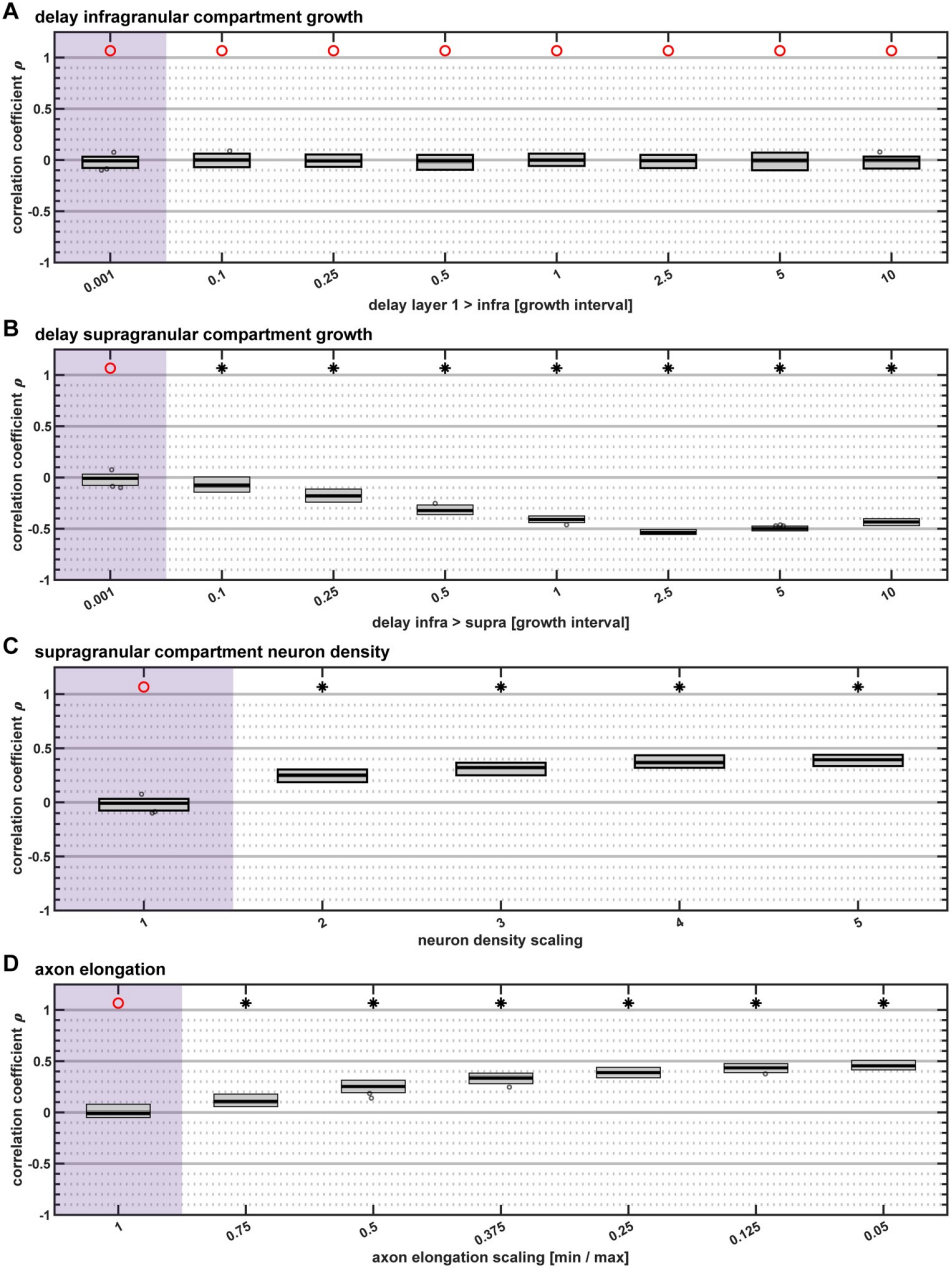
Correlation of supragranular contribution with neuron density difference. Spearman rank correlation coefficients for the correlation between the supragranular contribution of a projection and the neuron density difference between the connected areas. We used a sign test to determine whether the distribution of associated Spearman rank correlation p-values had a median value smaller than α = 0.05. The result of the sign test is indicated on top; black star: median p < 0.05, red circle: median p ≥ 0.05. (A) Delays in infragranular compartment growth did not affect the correlation. (B) As the growth of the supragranular compartment was increasingly delayed, a negative correlation between supragranular contribution and density difference emerged. (C) An increase in the density of the supragranular compartment relative to the infragranular compartment resulted in a positive correlation. (D) As the axon elongation scaling factor decreased and elongation values diverged, a positive correlation emerged. Box plots show distribution across 50 simulation instances per implementation, indicating median (line), interquartile range (dark grey box), data range (light grey box) and outliers (circles, outside of 2.7 standard deviations). Parameter values that correspond to baseline (i.e., with no feature implemented), are highlighted in purple.

When none of these four features was included in the model, that is, it was implemented at baseline settings, there was no correlation between the relative density of connected areas and the supragranular contribution to the projection linking them (Fig 3). Instead, for source areas of all neuron densities and connections across all density differences, the distributions of the contribution from the supragranular compartment (*N*_SG_%) were similar, with a mean around 50% and about equal variances (cf. S1 Fig, rows highlighted in purple), indicating that there was no preferential connection formation of either laminar compartment across areas or density differences. Moreover, the extension of the *in silico* model by laminar compartments did not affect the characteristics of inter-areal connection existence (i.e., considering only the binary status of connections as absent or present) reported previously for the *in silico* model [22]. We still observed a negative correlation between the neuron density of areas and the number of connections they maintain (area degree) (cf. S2 Fig), consistent with empirical observations [13,14]. The application of classifiers, that were trained on the simulated networks to predict connection existence from relative differentiation and spatial proximity, to empirical data also resulted in the good classification performance reported previously (cf. S3 Fig), indicating that our *in silico* model captured *in vivo* relationships between architecture, spatial proximity and connectivity.

### Delay in infragranular compartment growth did not affect laminar projection patterns

When the *in silico* model implementation included a delay between the time of origin of the layer 1 compartment and the infragranular compartment (with the supragranular compartment being formed at the same time as the infragranular compartment) within individual areas, we did not observe changes in the laminar patterns of projections origins relative to the implementation of the model at baseline settings (Fig 3A and S1A Fig). However, at very long delays, both the magnitude of the negative correlation of area density with area degree (S2A Fig) and the simulation-to-empirical classification performance (S3A Fig) decreased, indicating that the simulated network became less similar to empirical connectomes with respect to characteristics of connection existence.

### Delay in supragranular compartment growth resulted in negative correlation of supragranular contribution with relative differentiation

Inclusion of a delay between the time points of neurogenesis of the infragranular compartment and the supragranular compartment within an individual area substantially changed the laminar patterns of projection origins, resulting in a negative correlation between the relative density of connected areas and the supragranular compartment contribution to projections (Fig 3B). The longer the time between the formation of the infragranular compartment and the supragranular compartment of areas, the stronger this negative correlation became. Moreover, at longer delays, the magnitude of the negative correlation of area density with area degree (S2B Fig) as well as the simulation-to-empirical classification performance (S3B Fig) decreased, again indicating that the simulated network became less similar to empirical connectomes with respect to characteristics of connection existence.

### Unequal opportunities to connect affected laminar projection patterns

To explore how the negative correlation between density difference and supragranular compartment contribution emerged as the temporal delay between the compartments increased, we considered the distributions of *N*_SG_% values for source areas of different neuron densities and connections across different density differences (S1B Fig). As the delay increased, the distributions changed from their uniform appearance at baseline settings and became strongly skewed. At the longest delays, the supragranular contribution from areas of low neuron density increased as they connected to areas of successively higher neuron density (i.e., as the density difference to the target area became smaller), with connections to areas of similar neuron density constituted largely by infragranular compartment neurons. In contrast, this pattern shifted for areas of high neuron density, where connections to areas of similar neuron density arose evenly from both the infragranular and the supragranular compartment while connections to areas of lower neuron density became successively more dominated by the infragranular compartment as the density difference to the target area increased. We suggest that these changes in laminar patterns of projection origins arose from consequences of the delay in supragranular compartment growth as follows: Neurons in areas of lower neuron density, which were the first to appear on the cortical sheet, started connecting relatively early, while not all target areas were available, and therefore connected more frequently to areas of similar neuron density. But at longer delays, this applied only to the infragranular compartment, because the supragranular compartments in areas of lower neuron density grew after a large portion of the cortical sheet had already appeared, affording them the opportunity to connect across a wide range of density differences. Hence, in low-density areas, connections to higher-density areas (i.e., across larger negative density differences) originated predominantly in the supragranular compartment, while connections to areas with more similar density originated predominantly in the infragranular compartment. This pattern shifted for high-density areas, which grew after the low-density areas. Here, the infragranular compartment neurons had the opportunity to connect to most other areas, which were already present when these neurons appeared. However, at large delays in supragranular compartment growth, these infragranular neuron axons travelled across a cortical sheet that was not populated with supragranular neurons yet, increasing the range the neurons’ axons were likely to traverse before encountering a target neuron (relative to baseline settings). Supragranular neurons in high-density areas, in contrast, appeared once all other neurons had grown, making them as likely to encounter target neurons in other areas as at baseline. Thus, in high-density areas, infragranular compartment neurons were more likely to reach areas of less similar neuron density than supragranular neurons, resulting in larger infragranular compartment contribution for connections across relatively large density differences. As these unequal opportunities to connect, which areas of different neuron density and their laminar compartments encountered, combined, they resulted in an overall negative correlation between supragranular compartment contribution and density difference as the delay of supragranular compartment growth increased.

### Scaling of supragranular density did not result in representative laminar patterns of projection origins

Another feature that substantially affected the laminar patterns of projection origins was a scaling of the neuron density of the supragranular compartment relative to the infragranular compartment. As the relative density became larger, a successively stronger positive correlation between the relative density of connected areas and the supragranular compartment contribution to projections resulted (Fig 3C). The first characteristic of connection existence which we considered here, namely the negative correlation of area density with area degree (S2C Fig), was only negligibly affected by a change in the supragranular compartment neuron density parameter value. The second characteristic of connection existence, the simulation-to-empirical classification performance measured as Youden index *J* (S3C Fig), progressively increased by less than 0.1 across the range of tested parameter values.

### Supragranular contribution increased due to an increase of relative neuron numbers in the supragranular compartment

To identify the source of the positive correlation between density difference and supragranular compartment contribution which emerged as the relative density of the supragranular compartments increased, we again considered the distributions of *N*_SG_% values for source areas of different neuron densities and connections across different density differences (S1C Fig). As the relative neuron density increased from areas of lower neuron density to areas of higher neuron density, and as this divergence became stronger with an increasing scaling parameter value, the distributions of the *N*_SG_% values shifted upwards, away from the balanced contribution observed at baseline settings and for areas of the lowest neuron density where infragranular and supragranular compartments were of equal neuron density across the whole range of parameter values. However, this effect arose exclusively at the level of source areas. The distribution of *N*_SG_% values was uniform for connections to all target areas (i.e., across all density differences) within source areas of a given neuron density, indicating that the fraction of supragranular contribution was unaffected by relative differentiation between connected areas. Since areas of lower neuron density necessarily formed the connections across the smallest density differences and areas of higher neuron density correspondingly were the source of connections with the largest density differences, the aggregate profile of *N*_SG_% values exhibited a systematic increase of *N*_SG_% with density difference. In sum, the positive correlation observed for model implementations with pronounced differences between relative neuron density across areas resulted from the fact that areas of different neuron density had uniform profiles of *N*_SG_% values for all their connections, which, however, differed between areas. It did not result from a graded pattern of *N*_SG_% values found within any one area.

**Fig 4:**
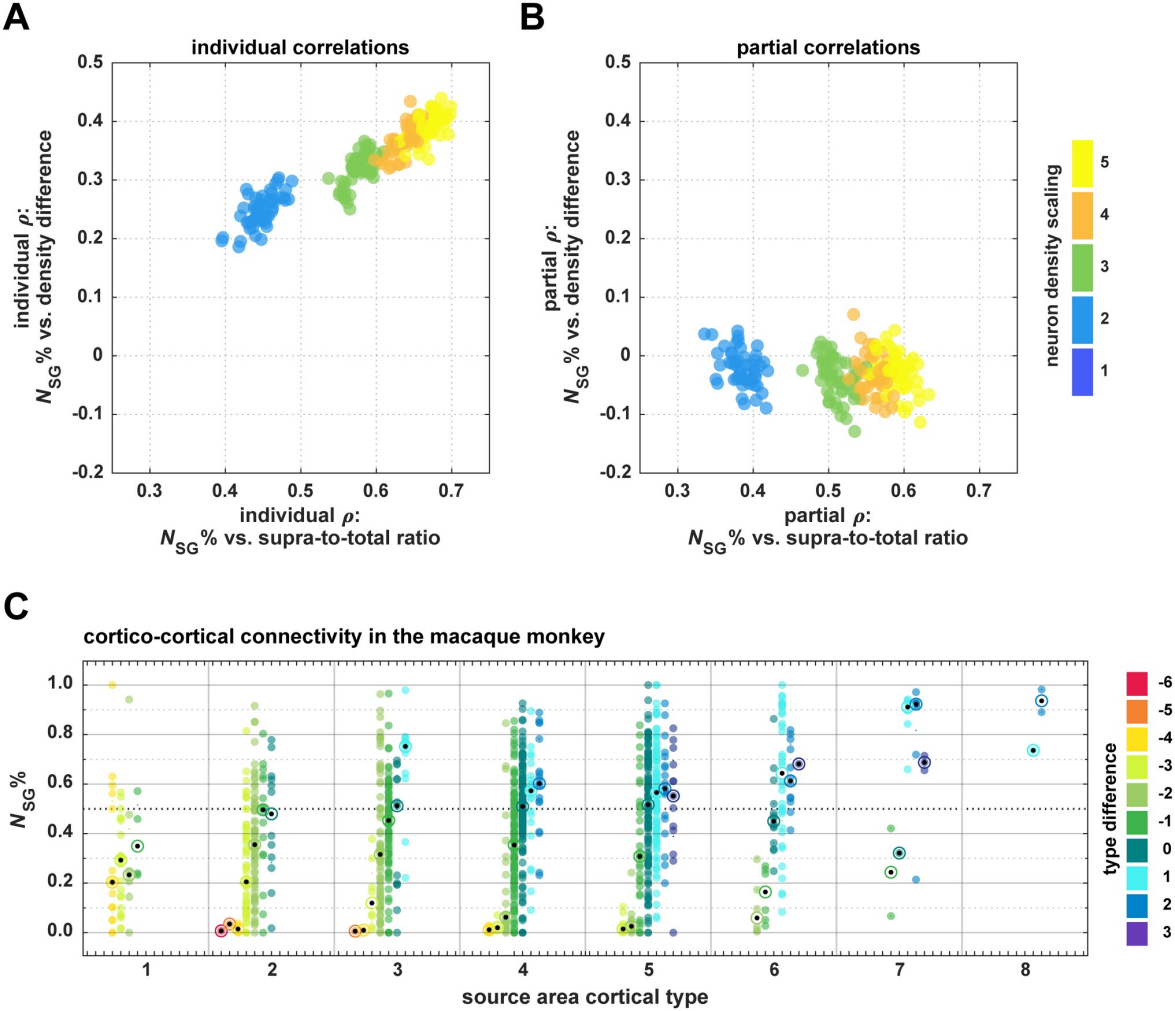
Neuron density scaling did not result in realistic laminar patterns of projection origins. (A, B) The positive correlation observed for increasing scaling of supragranular compartment neuron density is abolished by controlling for the number of supragranular neurons. Individually, both density difference and the ratio of supragranular neurons to total neurons are increasingly correlated with supragranular contribution as the density scaling factor increases (A). However, the correlation of density difference with supragranular contribution decreases to baseline levels when it is included alongside supra-to-total neuron ratio in a partial correlation, while correlation values for supra-to-total neuron ratio are hardly affected by the inclusion of density difference (B). Note that no correlation coefficients are shown for a scaling factor of 1, the baseline value, because here all supra-to-total ratios are 0.5 and no correlation can be computed. (C) In the macaque monkey, supragranular contribution is distributed across differences in architectonic differentiation (see color scale) in a graded manner even within areas of a particular level of differentiation, contrary to the distributions observed in our simulation experiments (cf. S1C Fig). Projections are grouped according to the cortical type of their source area and the type difference between the connected areas (see color scale) and for each column a median is indicated (target). *N*_SG_% values from [73], cortical type values from [15].

To demonstrate that the positive correlation was mediated exclusively by the increased number of supragranular neurons, we computed a partial correlation of supragranular compartment contribution with relative neuron density of connected areas while controlling for the ratio of supragranular compartment neurons to total neurons in the source area. In individual correlations with supragranular compartment contribution, both measures (density difference and neuron ratio) exhibited a strong positive correlation (Fig 4A). However, if all three measures were included simultaneously in a partial correlation, the correlation between density difference and supragranular contribution was abolished, while the correlation between neuron ratio and supragranular contribution was affected only negligibly (Fig 4B). (See S4 Fig for the third aspect of this partial correlation, the correlation between density difference and the ratio of supragranular compartment neurons to total neurons in the source area.) Hence, as the value of the supragranular compartment neuron density scaling parameter became larger, the difference in neuron numbers between infragranular and supragranular compartments increased, and became especially pronounced for areas of higher neuron density. This increase in supragranular neurons accounts for the increase in *N*_SG_% values in the supragranular compartment at higher values of the supragranular density parameter and is the factor that determines the correlation between density difference and supragranular compartment contribution.

### In vivo, the positive correlation of relative differentiation with laminar patterns of projection origins does not result from a relative increase in supragranular neuron density

**Fig 5:**
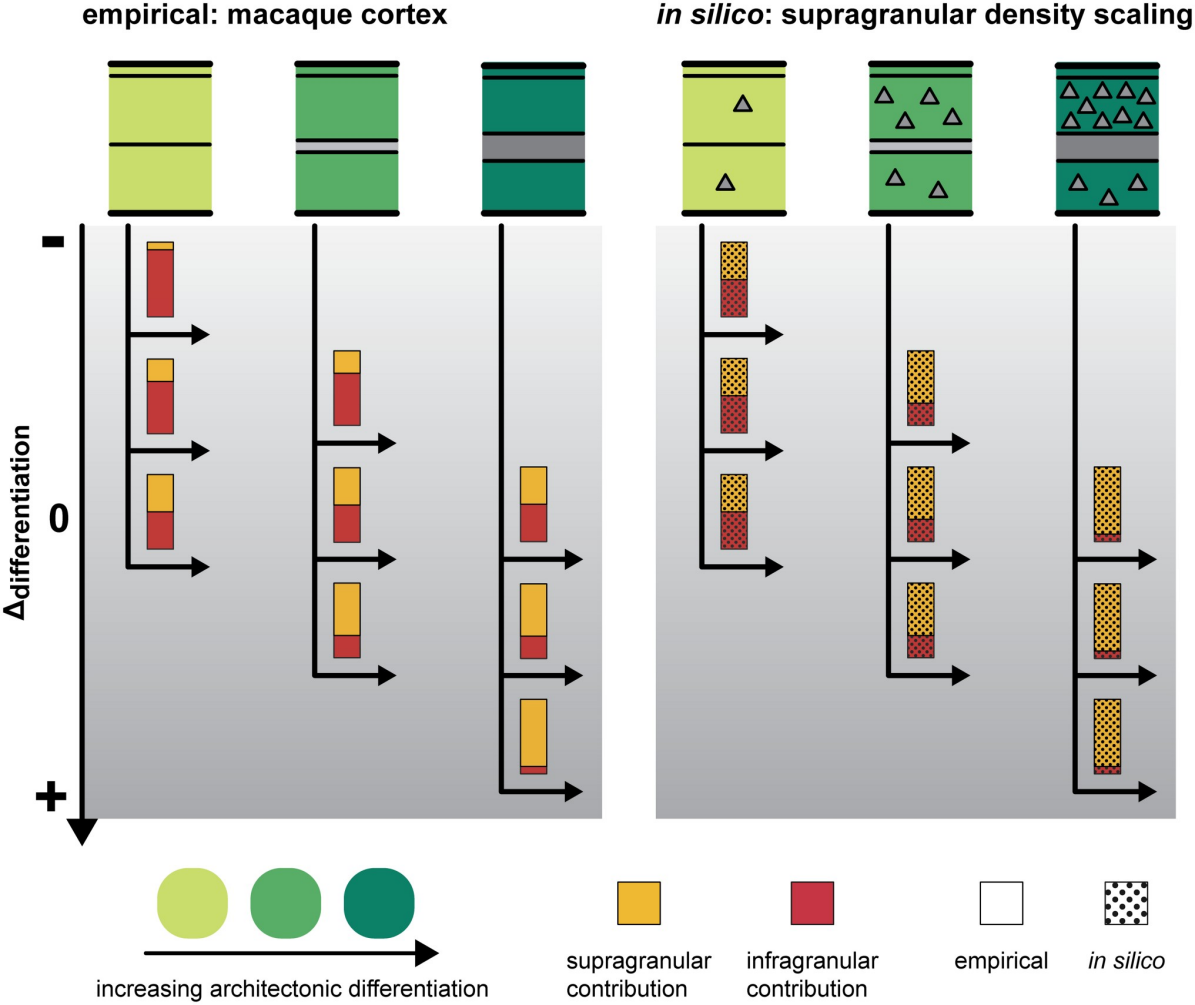
Supragranular contribution did not vary across density differences for supragranular neuron density scaling. As the scaling parameter of supragranular neuron density increased, a positive correlation between relative differentiation and supragranular contribution emerged. However, when this feature was implemented, the profile of supragranular contribution values across density differences did not match the profile observed in empirical data. In the macaque cortex, connections from a single area show a wide range of laminar patterns (Fig 4C). Depending on which target area the connection leads to, the origin shifts from more infragranular to more supragranular. In our simulation experiments, each source area had a characteristic value of supragranular contribution, and all its connections shared this value, independent of the target area. In conjunction, these static values then looked like a positive correlation because, by definition, the beginning and end of the spectrum of difference in differentiation contain only connections from areas of very low and very high neuron density (areas with low neuron density cannot form connections with large positive values of density difference, because density difference is calculated as density _source area_−density _target area_; the same logic holds for connections from areas with high neuron density, which cannot have negative values of density difference). Areas with higher neuron density had a larger fraction of their neurons in the supragranular compartment, and therefore their connections originated mostly from this compartment. We could show that the shift in the origin patterns was only due to this relative increase of neuron density in the supragranular compartment by regressing out the ratio between the number of supragranular neurons to the total number of neurons in each area (Fig 4B). When we accounted for this difference between areas, the correlation between supragranular contribution and relative differentiation vanished.

In contrast to the results presented here for our *in silico* model, the positive correlation between supragranular contribution and relative architectonic differentiation of connected areas which is observed in mammalian cortices is not the result of a combination of distributions of supragranular contribution that differ between source areas, but are uniform within source areas of a given differentiation. Instead, individual areas across the whole spectrum of architectonic differentiation exhibit the graded pattern of supragranular contribution increase that has been reported for the aggregate connectomes. In Fig 4C, we show this in the supragranular contribution to cortico-cortical connections of the macaque brain reported by [73], with connections grouped according to the architectonic differentiation of the source areas (using the qualitative ranking measure of cortical type [15]). The profiles of *N*_SG_% values differ markedly from the uniform distributions observed in similar plots of the simulated networks. This discrepancy is illustrated in Fig 5.

### Divergence in axon elongation resulted in laminar patterns of projection origins that exhibited the empirically observed relation to relative differentiation

Lastly, we introduced a divergence in the elongation of axons, where distances travelled by axons during each time step differed between infragranular and supragranular compartment neurons and gradually shifted along with neuron density, mirroring the phenomenon of changing relative cell sizes observed across the mammalian cortex (externopyramidization). With increasing divergence in axon elongation, we observed a stronger positive correlation between supragranular contributions to connections and relative differentiation of connected areas (Fig 3D). Taking into account the distributions of *N*_SG_% values across connections of varying density difference and for source areas of different neuron density (S1D Fig), it became apparent that the *N*_SG_% values differed according to density difference and moreover became more unequal as the divergence in axon elongations between the slowest and the fastest growing laminar compartments increased. Hence, varying the axon elongation affected the correlation between supragranular compartment contribution and relative differentiation at the level of individual areas, as shown in the macaque monkey (Fig 4C), and not only in aggregate across all areas, as shown for the scaling of supragranular compartment neuron density (S1C Fig).

Moreover, the first considered characteristic of connection existence, that is, the negative correlation of area density with area degree (S2D Fig), remained essentially unaffected by the changes in axon elongation. The second characteristic of connection existence, the simulation-to-empirical classification performance measured as Youden index *J* (S3D Fig), progressively decreased by less than 0.1 across the range of tested parameter values.

The variation of *N*_SG_% across density differences became apparent mostly for high-density areas. Low-density areas were formed early in the simulation and their neurons were mostly connected by the time the high-density areas were formed. The effect of restricting axon elongation did not become as apparent as for low-density areas, because the faster growing infragranular neurons did not yet have the entire cortical sheet to grow into, as the faster growing supragranular neurons in high-density areas did. This entailed that the infragranular neurons could not manifest their full potential for growth, and inequalities in the contribution of the two laminar compartments could not form as easily.

### Spatio-temporal interactions were a necessary condition for the emergence of the positive correlation between laminar patterns and relative differentiation

**Fig 6:**
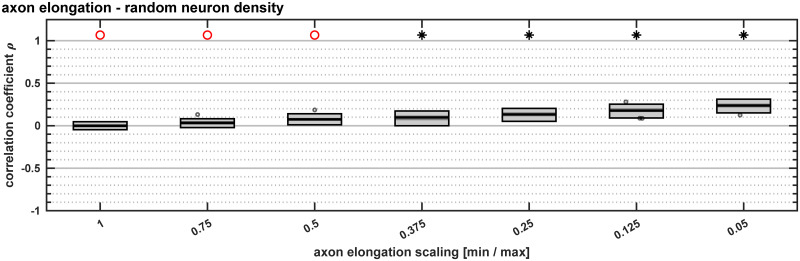
Abolishing the ordered neuron density gradient decreased the positive correlation observed for axon elongation scaling. Spearman rank correlation coefficients for the correlation between the supragranular contribution of a projection and the neuron density difference between the connected areas. Here, we implemented the scaling in axon elongation but assigned area neuron densities randomly across times of origin, thus removing the ordered gradient of areas with higher neuron density forming at later points in time that was present in the other implementations. We used a sign test to determine whether the distribution of associated Spearman rank correlation p-values had a median value smaller than α = 0.05. The result of the sign test is indicated on top; black star: median p < 0.05, red circle: median p ≥ 0.05. Box plots show distribution across 50 simulation instances per implementation, indicating median (line), interquartile range (dark grey box), data range (light grey box) and outliers (circles, outside of 2.7 standard deviations).

If the correspondence between time of origin and architectonic differentiation was removed, the effects reported for diverging axon elongations became weaker. This could be concluded from implementations of the *in silico* model in which the neuron density of areas was drawn randomly from the set of neuron densities present in the baseline setting. We simulated 50 instances of such a random variation of area neuron density with time of origin for each parameter value at which we evaluated the feature of axon elongation. Here, as divergence in axon elongation increased, correlation between supragranular compartment contribution and density difference increased slightly, but remained at small magnitudes even for large divergences (Fig 6). That any correlation appeared at all was due to the restricted ranges of neurons in specific laminar compartments, causing projections from low density and high density areas to be formed primarily from the infragranular and supragranular compartment, respectively. The thereby reduced ranges of *N*_SG_% values for these areas, which constituted the source areas for projections at the very low and very high end of the density difference scale, respectively, then combined to an apparent positive correlation of supragranular contribution with density difference across all areas (similar to the effect observed for the scaling of supragranular compartment neuron density). Moreover, when the neuron density of an area was not correlated with the area’s time of neurogenesis, there resulted a positive correlation between neuron density and area degree (S5A Fig), contrary to what has been observed empirically [13,14]. In addition, simulation-to-empirical classification performance was dramatically reduced (S5B Fig) and became quite variable, indicating that it depended strongly on the concrete random layout of neuron densities that was realized in a particular simulation instance.

### Combinations of features

If any two of the previously presented features (delay in infragranular compartment growth, delay in supragranular compartment growth, scaling of supragranular compartment neuron density, axon elongation) were combined in the *in silico* model, no unexpected effects emerged from their co-occurrence (S6 Fig). Instead, the previously described effects superimposed in a straight-forward manner, specifically, a delay in supragranular compartment growth resulted in an increasingly negative correlation between supragranular compartment contribution and relative differentiation, caused by the aforementioned mechanisms. Including a delay in infragranular compartment growth did not modulate the effects caused by the other two features. The inclusion of an increase in the supragranular density scaling parameter value resulted in a relative increase in the correlation between supragranular compartment contribution and relative differentiation, but this correlation was again abolished by controlling for the number of supragranular neurons. Finally, including diverging levels of axon elongation resulted in a relative increase in this correlation. If all four features were implemented simultaneously (S7 Fig), the superposition ensued as expected for the three features of delay in supragranular compartment growth, scaling of supragranular compartment neuron density and axon elongation. Again, the effect of supragranular compartment neuron density scaling was abolished by controlling for the number of supragranular neurons (S7B Fig). At longer delays in infragranular compartment growth, correlation coefficients were comparatively higher, presenting an effect of this delay that was not observed in the other feature combinations. Given that this effect did not occur in any of the pair-wise feature combinations and was not affected by the scaling in supragranular compartment neuron density (since it persisted regardless of whether the supra-to-total neuron ratio was taken into account), it appears to be specific to the combination of the three features of the two delays and axon elongation. Since including the delay in supragranular compartment growth resulted in a negative correlation between supragranular contribution and relative differentiation, the interaction was not sufficient to increase the correlation coefficient relative to the inclusion of only axon elongation (Fig 3D).

## Discussion

The architectonic type principle has far-reaching implications for the understanding of the organization of structural connectivity in the mammalian brain [3]. However, it remains unclear how the architectonic type principle emerges. Since detailed experimental observations of developmental events, which could answer this question, are difficult to obtain, simulation experiments are the most feasible way to systematically evaluate hypotheses about the mechanisms that underlie the emergence of the architectonic type principle. Previously, we reported that realistic assumptions about the spatio-temporal patterns of neurogenesis included in an *in silico* model can lead to simulated networks that comply with the regularities that are described by the architectonic type principle in the mammalian cortex with respect to the existence of connections between areas [22]. Here, we extended this *in silico* model by laminar compartments to probe not only the existence of connections, but also the distribution of the connecting neurons across layers, that is, the laminar patterns of projection origins. Moreover, we introduced four features, three of which changed the spatio-temporal patterns of neurogenesis: a delay in the growth of the infragranular compartment (its time point of neurogenesis was delayed relative to the time point of neurogenesis of layer 1), a delay in the growth of the supragranular compartment (its time point of neurogenesis was delayed relative to the time point of neurogenesis of the infragranular compartment) and a scaling in the relative neuron density of the supragranular compartment. The fourth feature, in contrast, affected cell-intrinsic properties by changing the axon elongation per time step according to a neuron’s laminar compartment and an area’s architectonic differentiation. By varying the strength (i.e., the parameter value) with which each of the four features was included in the *in silico* model, we tested the sensitivity of the laminar projection patterns to a given parameter. Moreover, we combined the features to test for emergent effects that could not be observed by including the four features individually.

### Spatio-temporal interactions could not be shown to produce empirically observed patterns of laminar projection origins

Including the three spatio-temporal features in the *in silico* model did not induce the simulated networks to exhibit the empirically observed patterns of projections origins. A delay in the growth of the infragranular compartment did not affect the laminar patterns at all (Fig 3A), while a delay in the growth of the supragranular compartment resulted in a negative correlation between supragranular contribution to connections and relative differentiation (Fig 3B), which is the opposite of what has been observed in the mammalian cortex. This negative correlation indicated that areas of lower density formed their connections increasingly from the supragranular compartment the more pronounced the difference in neuron density to the target area became, while areas of higher density formed their connections increasingly from the infragranular compartment the larger the difference in neuron density became. This effect was due to unequal opportunities to connect that neurons in the infragranular and supragranular compartments of lower and higher density areas encountered. A combination of both delays, which is the model implementation that most closely resembles the radial gradient of neurogenesis observed *in vivo* [40,41], did not result in unexpected effects. Instead, the effects of both delays superimposed without any interactions. Since the delay in infragranular compartment growth did not affect laminar patterns of origins, this means that the results for a combination of both delays were indistinguishable from the results obtained from including the delay in supragranular compartment growth individually (S6A Fig).

Although including a scaling of supragranular compartment neuron density did result in a positive correlation between supragranular contribution and relative differentiation for larger parameter values (Fig 3C), this correlation was not accompanied by a graded distribution of supragranular contributions across density differences between connected areas (S1C Fig). Instead, source areas of each neuron density level formed their connections at a characteristic supragranular contribution, which did not differ for connections across different density differences (Fig 5). The positive correlation emerging overall thus results from the fact that by definition areas of lower neuron density form projections across the smallest neuron density differences and areas of highest density form projections across the largest neuron density differences. This composite correlation is in stark contrast to the patterns of supragranular contribution that have been observed empirically, where areas of each level of architectonic differentiation exhibit a graded pattern of supragranular contributions that varies with the difference in architectonic differentiation to the target area (Fig 4C). Moreover, the aggregate positive correlation could be abolished by controlling for the ratio of supragranular neurons to total neurons in the source area (Fig 4B). This implies that the shifts that occurred to the distributions of supragranular compartment contribution for each level of source area neuron density (which were uniform across density differences) was caused by the preponderance of supragranular compartment neurons compared to infragranular compartment neurons at larger values of the supragranular compartment neuron density scaling parameter. The scaling of supragranular neuron density would be expected to be a less relevant factor in the rodent brain, where levels of architectonic differentiation do not generally diverge as strongly across the cortex as they do, for example, in the primate cortex. This prediction is indeed confirmed by recent comprehensive connectivity data for the mouse brain, which show less distinctive laminar projection patterns [74]. The applicability of the architectonic type principle, in general, hinges on the presence of gradients in architectonic differentiation (discussed in [75]).

Summing up, in our simulation experiments it was not sufficient to modify spatio-temporal patterns of neurogenesis in order to produce simulated networks in which the origins of connections were distributed across laminar compartments in a manner that was similar to the patterns observed empirically in the mammalian cortex.

### Differences in cell-intrinsic properties that were linked to architectonic differentiation produced realistic patterns of projection origins

We introduced graded differences in a property that was intrinsic to individual neurons, namely the elongation of their axon per time step. This property was changed in accordance with an area’s neuron density, such that the divergence in axon elongation between the neurons in the infragranular and in the supragranular compartment varied systematically along the gradient of architectonic differentiation (represented by neuron density). Similar to the changes in relative cell size between the infragranular layers and the supragranular layers that have been described as externopyramidization [61,62] and that also vary systematically with architectonic differentiation (reviewed in [63]), we varied the relative levels of axon elongation across laminar compartments and areas. At larger levels of divergence a positive correlation between supragranular compartment contributions and relative differentiation emerged (Fig 3D). This correlation mirrored empirically observed patterns of supragranular compartment contributions, as it was realized across connections of differing density differences already at the level of individual areas (S1D Fig). Moreover, for the emergence of this positive correlation, the spatio-temporal patterns of neurogenesis, which were previously identified to be sufficient for the emergence of realistic patterns of connection existence [22], had to be present. When the underlying relationship of time of origin to areas’ neuron density (i.e., higher neuron density with later time of origin) was removed, the positive correlation between relative architectonic differentiation and supragranular contribution became weaker (Fig 6).

**Fig 7:**
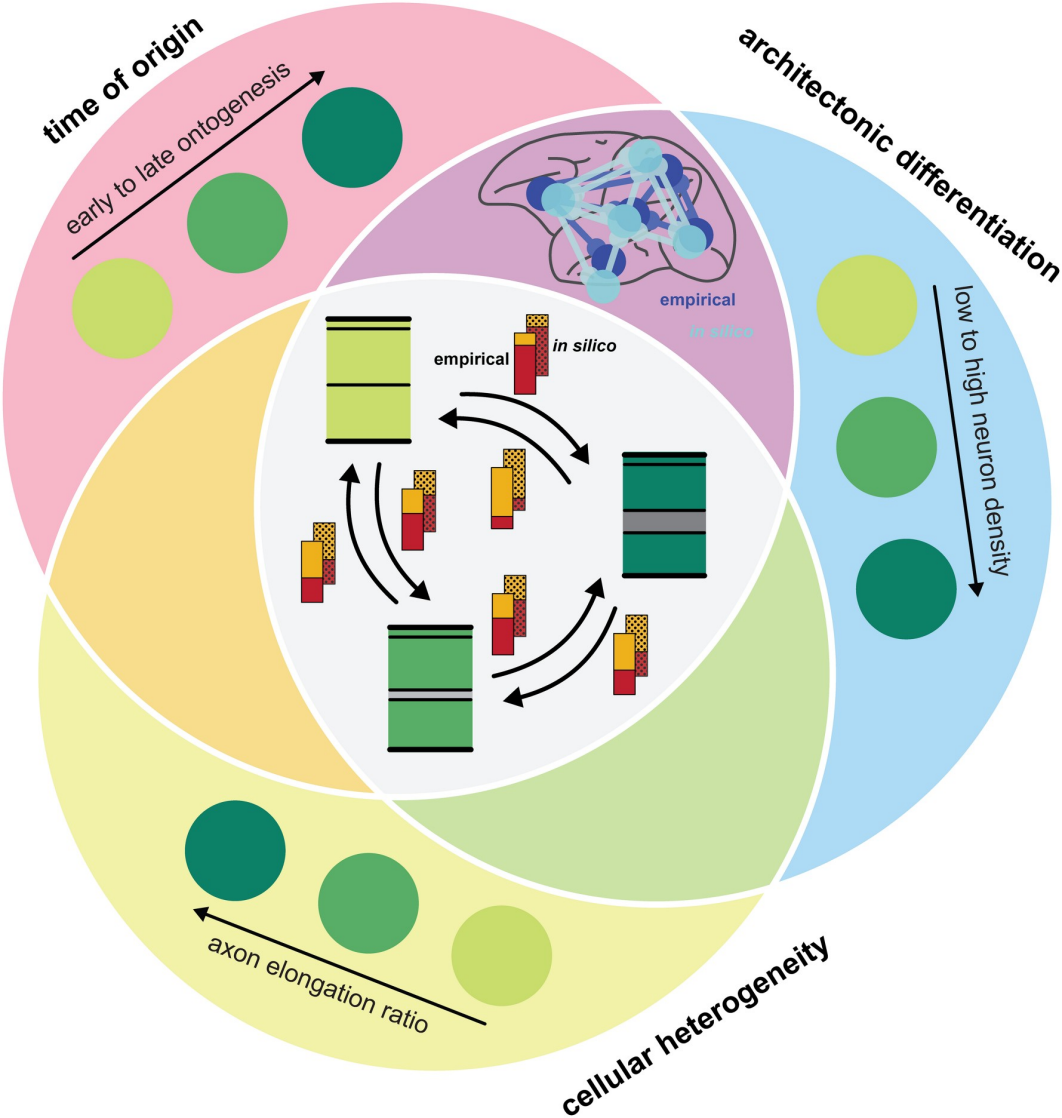
Realistic laminar patterns can arise from an interaction of spatio-temporal gradients in neurogenesis with gradients in cell-intrinsic properties. Our *in silico* model of the developing cortical sheet included three properties of areas which changed gradually. These were time of origin (red), architectonic differentiation (blue) and cellular heterogeneity, changing the balance of axon elongation in the infragranular compared to the supragranular compartment (yellow). Axon elongation values changed gradually, from larger in the infragranular compartment to larger in the supragranular compartment, yielding an increasing ratio of the supragranular to infragranular value. While realistic patterns of connection existence can arise from an alignment of the two gradients of time of origin and architectonic differentiation [22] (purple), the inclusion of a gradient in the cell-intrinsic property of axon elongation, which was aligned to the gradient of architectonic differentiation, was crucial for the emergence of realistic laminar patterns of projection origins (gray). Bars indicate laminar contributions to projections, with red representing contribution from the infragranular compartment and orange representing contribution from the supragranular compartment. Solid colors indicate empirically observed relationships, captured by the architectonic type principle, while dotted colors indicate simulated patterns.

Thus, in our simulation experiments, differences in cell-intrinsic properties that varied with architectonic differentiation and spatio-temporal patterns of neurogenesis interacted, allowing the formation of simulated networks that exhibited a relationship of laminar patterns of connection origins to relative differentiation of connected areas which resembled their relationship observed in the mammalian cortex (Fig 7).

### Differences in cellular properties *in silico* and *in vivo*

From our simulation experiments it appears that the establishment of laminar patterns of projection origins does not easily arise from spatio-temporal interactions in the developing cortical sheet. More specific assumptions about developmental processes were necessary for our *in silico* model to generate realistic laminar projection patterns. By modifying the cell-intrinsic property of axon elongation, we introduced differences between individual neurons, which were modelled to vary systematically with the architectonic differentiation of areas and which also modified properties of infragranular compartment neurons and supragranular compartment neurons separately. This approach is consistent with a wealth of observations in the mammalian cortex, demonstrating that many properties of neurons vary both with architectonic differentiation and laminar position. For example, myelination, cellular markers of synaptic stability and plasticity, cellular morphological properties, the distribution and density of neurotransmitter receptors as well as the density of neurons expressing parvalbumin and calbindin have all been described to change across the spectrum of architectonic differentiation [7,17,56–60]. Similarly, the expression of many transcription factors and neurotransmitter receptors as well as the distribution of neurons expressing proteins such as parvalbumin, calbindin, calretinin and latexin have been shown to vary across cortical layers [7,58,76–79], as have physiological [80–83] and histochemical properties of pyramidal neurons [84,85]. Moreover, there is ample evidence that axons are guided by attractants and repellants both on large spatial scales, for example during the establishment of contralateral or cortico-spinal projections [86–88], and on small spatial scales, for example during the specification of laminar projection targets [89–92]. These processes are affected by a multitude of diffusible and membrane-bound molecules [93], and an additional layer of complexity is added by the fact that the same guidance molecule can have opposing effects on different neurons, depending on the receptor complement that is expressed by the guided neurons [94,95]. Given this large range in spatial scales documented for axon guidance mechanisms, it appears plausible that similar mechanisms could cover the whole range of spatial scales, that is, also the mesoscopic, medium spatial scale of ipsilateral cortico-cortical connectivity, thus affecting laminar patterns of projection origins.

Our implementation of differences in cellular properties assumed the existence of two opposing trends in axon elongation, which changed in opposite directions along the spectrum of architectonic differentiation in the infragranular and supragranular compartments. It is conceivable that gene expression patterns across the mammalian cortex could, in a similar manner, mediate differences in axon elongation and thereby the growth speed of axons across the differentiation spectrum and cortical layers, and hence, that our chosen experimental manipulation would mirror an actual mechanism occurring in the mammalian brain. However, this is not the premise under which we interpreted our *in silico* model. Rather, we wanted to probe how the pattern of two opposing gradients, varying the properties of neurons along the gradient of architectonic differentiation separately in the two laminar compartments, affected laminar patterns of projection origins. We chose to modify the elongation of axons, because we could model this property to follow the pattern of shifting relative cell sizes described by the phenomenon of externopyramidization [61,62]. As such, axon elongation was a useful proxy which we chose out of other possible cellular properties that may differ across the spectrum of architectonic differentiation and between cortical layers, such as dynamics of axon outgrowth (waiting periods in the white matter [96–98], pruning of synapses [98–100]) or specificity in synapse formation upon contact with potential target neurons. Another possibility to explore in future simulation experiments, shedding the simplification of completely stochastic axon outgrowth, would be the susceptibility of neurons to axon guidance mechanisms. Based on the fact that architectonic differentiation goes along with marked differences in the presence and laminar distribution of specific cell types and gene expression patterns, as described above, such axon guidance could operate both on a general level, affecting axons’ attractedness to or repulsion from areas based on their degree of architectonic differentiation, and on a more specific level, affecting projection patterns towards specific neuron populations.

Although the assumption of two opposing gradients in the infragranular and supragranular compartments may appear bold at first, it is plausible when considering related experimental observations. Concerted changes in macroscopic and microscopic architectonic features mirroring architectonic differentiation are pervasive in the adult mammalian cortex, as described in the preceding. It has been demonstrated that common gene expression signatures can distinguish neuron subtypes and regional identity, which supports a transcriptional basis for differences in cortical cytoarchitecture [101–103]. Obviously, these gradients arise from developmental mechanisms [3]. For example, the time point at which a neuron is formed is flagged by markers of embryonic age and impacts the trajectory of differentiation the neuron follows [104]. The existence of similar gradients prior to the finalization of adult levels of differentiation is difficult to observe experimentally, but does not appear contentious. Is it plausible, then, that two opposing gradients should exist in the infragranular and the supragranular layers? Distinct molecular mechanisms have been identified that are crucial in the specification of infragranular and supragranular neurons (reviewed in [105]). Moreover, infragranular and supragranular neurons have been reported to exhibit diverging developmental time courses [98,106,107], for example in the modification of their spatial divergence or whether they display waiting periods. These observations demonstrate that the two laminar compartments are sufficiently dissociated in their specification to support opposing gradients, for example in their sensitivity to axon guidance molecules, across the architectonic differentiation spectrum. Interestingly, from an evolutionary perspective, more differentiated areas are newer than less differentiated areas [3,46], and increased differentiation is mediated by lengthening developmental schedules, which result in an increase in neuron complement especially in the supragranular layers [54,55]. This specific expansion would have opened up the supragranular neurons as a new substrate for connecting newly specified areas and for modification independent of pre-existing circuits involving infragranular neurons. Indeed, the increased prominence of supragranular layers has been suggested to be one of the crucial substrates for evolutionary adaptation in primates [108].

Our simulation experiments evaluated the laminar patterns of projection origins, but did not address how projection terminations were distributed across cortical layers. Termination patterns have also been shown to relate to relative differentiation of connected areas [5,15] and thus fall within the scope of the architectonic type principle. Further simulation experiments could probe which mechanisms possibly mediate their specification.

## Conclusion

The architectonic type principle conceptualizes structural connections between brain areas in terms of their relative architectonic differentiation, providing a mammalian-general principle for the organization of cortico-cortical connections [2–4]. How the empirically observed relationship between cortical architecture and features of connectivity emerges has not yet been elucidated by developmental studies, but it has been suggested to result from spatio-temporal interactions during neurogenesis [1,2,7,15]. Concerning the existence of connections, this mechanistic explanation is supported by results from simulation experiments [22,23]. Here, we expanded an *in silico* model of the developing cortical sheet to include laminar compartments and probed which factors might shape the laminar patterns of projection origins. Our results indicate that while the emergence of typical laminar patterns is indeed affected by spatio-temporal interactions during neurogenesis, the specifics of where and when neurons are formed are not the exclusive determinants of laminar patterns. A further specification of neuron identity, varying a cell-intrinsic property across the gradient of architectonic differentiation, was sufficient to enable our *in silico* model to generate realistic laminar patterns of projection origins. This suggests that future research should consider the intricacies of how neuron identity is specified developmentally, to identify the mechanistic underpinnings of the architectonic type principle and thereby advance our understanding of how connectivity in the mammalian cortex is organized.

## Methods

We simulated how cortical neurons in a single hemisphere and their interconnections may develop using a two-dimensional *in silico* model. The model has been described in detail in Beul et al. [22], and we summarize the main characteristics here, before describing the model extension in detail. On a two-dimensional plane, neuron somata develop and are assigned to cortical areas ([22], their Fig 2). Neurons belonging to a single area grow simultaneously, with sets of areas growing sequentially. Cortical areas are designed to be of the same size but to exhibit a range of neuron densities (i.e., number of neurons per area), therefore neuron numbers differ between areas (see [22] for more details). The specifics of where and when neuron somata develop are aligned to empirical neurodevelopmental findings and have been extensively tested in a previous report [22]. Here, we employed the model settings that were shown to yield the most realistic connectivity and that correspond most closely to observations of actual cortical development in mammals (growth layout “1D-2row-2or” in [22]). Specifically, our *in silico* model was set to grow expansively around two neurogenetic origins, such that more recently formed areas separate earlier formed areas, increasing the spatial distance between them over time ([22], their Fig 2). Moreover, it was set to have a positive correlation between time of neurogenesis and neuron density, such that the earliest formed areas have the lowest neuron density and the areas that develop last have the highest neuron density.

Each neuron has one axon which grows by a specific length, at a random angle, at each time step. Once the axon tip comes sufficiently close to a neuron soma, a connection is formed. Connection formation thus happens concurrently with neuron development and can be characterized as stochastic. The *in silico* model explores, which network properties may emerge from the simplest possible mechanisms. Although it is indisputable that axon guidance mechanisms make some contribution to shaping connectivity, it is not evident at which scales (e.g., targeting of cortical area, laminar compartment, specific cell types or cellular compartments) the effect of axon guidance mechanisms could result in the empirically observed laminar patterns of connection origins that predictably vary with the relative architectonic differentiation of connected areas. Moreover, factors that cause an increase in growth rate do not necessarily orient growing axons [90], and the same axon guidance molecule may have different effects on different types of neurons [95] or on the same neuron at different points in time [87], so the specific action of axon guidance molecules can only be known with respect to a defined neuron type that is receptive to them at a defined time point. Considering these facts, a simple implementation of axon guidance mechanisms appears far-fetched. Under these circumstances, it appears to be a useful approach to first explore the capacity and limitations of random growth. Specific patterns that are not captured by this approach may then be ascribed to targeted growth mechanisms. Therefore, we did not incorporate any assumptions about specific mechanisms of axon guidance. Instead, we probe which properties may already result from stochastic connection formation alone. This approach is also motivated by evidence in rodents, which indicates that the initial outgrowth of axons is unspecific, with no particular spatial orientation [109].

After a fixed number of time steps, we evaluated the final state of the simulated cortical sheet. We extracted measures that were analogous to measures that were used in previous analyses in the mammalian cortex ([22], their Fig 1). These were the area-wise connectivity, the difference in neuron density between areas, and areas’ Euclidean distance on the simulated cortical sheet.

Here, to probe the origin patterns of cortico-cortical projections across cortical layers, we extended the previously used *in silico* model by a radial component, assigning the neuron somata to one of three laminar compartments (layer 1, supragranular compartment, infragranular compartment). The cortical sheet remained implemented in two dimensions, since we did not intend to model the growing out of axons towards the white matter or the laminar patterns of projection terminations. As we did previously, we evaluated the existence of projections between cortical areas. Additionally, we considered how the origins of projections were distributed across laminar compartments. Similarly to empirical studies, we report the fraction of projection neurons (for a given projection) which originated in the supragranular compartment, *N*_SG_%.

At the baseline setting, the neuron density of an area’s supragranular compartment was equal to the density of the infragranular compartment. Since there are generally few neurons in layer 1 [110,111], we chose a lower density of 15% of infragranular compartment density for layer 1. Moreover, since layer 1 is mainly a target for long-range projections (reviewed in [35]), we included layer 1 neurons in the *in silico* model only as connection targets, meaning they could form synapses with approaching axon tips, but they did not grow out axons themselves.

### Features implemented to modulate laminar projection patterns

We introduced four features that possibly affect how the origins of projections are distributed across laminar compartments and included these features in the *in silico* model individually or in conjunction (Fig 1). Three of these features changed the spatio-temporal pattern of neurogenesis, affecting where and when neurons develop. The fourth feature, in contrast, changed properties of the neurons themselves.

The first two features were temporal delays between the laminar compartments. *In vivo*, cortical neurons develop in an inside-out pattern (with the exception of layer 1 neurons, which develop first), where earlier born neurons come to reside in the lower cortical layers and later born neurons migrate upwards and become positioned successively closer towards layer 1 [40–43]. To simulate this radial gradient in time of neurogenesis within areas, we introduced two delay parameters, one for the delay between the time points of neurogenesis of layer 1 and infragranular compartment neurons and a second for the delay between the time points of neurogenesis of infragranular compartment neurons and supragranular compartment neurons. When one or both of the delay features were included in the *in silico* model, whole areas did not grow simultaneously any more, but instead laminar compartments appeared on the cortical sheet sequentially, with all the neurons of a laminar compartment appearing simultaneously.

The third feature we introduced was a scaling of the neuron density of the supragranular compartment. In the mammalian cortex, increases in overall neuron density across areas tend to be mediated mostly by increases in supragranular neuron density [54,55]. We therefore introduced a parameter that modified how much denser the supragranular compartment became relative to the infragranular compartment. While it left the variation in infragranular compartment density across areas unchanged from the baseline setting, this parameter determined to which level the relative density of laminar compartments increased for the highest infragranular compartment density. Supragranular compartment density was always equal to infragranular compartment density for the lowest infragranular compartment density and scaled up linearly in between these two extremes (areas of lowest to highest infragranular compartment density). For example, at baseline, that is with a supragranular compartment density scaling parameter value of 1, supragranular compartment density would be equal to infragranular compartment density for all areas. At a parameter value of 3, however, the density of the supragranular compartment would be three times the infragranular compartment density for the areas with the highest infragranular compartment density, while it would be double the infragranular compartment density for the areas with an infragranular compartment density halfway between lowest and highest infragranular compartment density.

The fourth feature, axon elongation scaling, did not affect spatio-temporal patterns of neurogenesis but modified properties of individual neurons while leaving their time and place of origin unchanged. As architectonic differentiation changes, so do properties of individual neurons, for example in morphological and physiological aspects [17,37]. One striking phenomenon is externopyramidization [61,62]: the relative sizes of cells in the laminar compartments shift with architectonic differentiation. Less differentiated areas tend to have their larger neurons in infragranular layers, but cells become more equal in size between infra- and supragranular layers for more differentiated areas, while very strongly differentiated areas, finally, tend to have their largest neurons in the supragranular layers. Evidence that larger cells are able to maintain longer connections (reviewed in [63]) indicates that cell-intrinsic properties play a role in shaping connectivity, even though the question of causality still remains. To generate differences in the likelihood that neurons will form long-range connections which arise from properties inherent to the neurons, we varied the elongation of axons, changing the distance they grow per time step, in a manner similar to the observed relative cell sizes. Neurons with larger axon elongation were predisposed towards longer connections, because they traversed a larger distance per time step and were therefore more likely to have travelled further before encountering a connection target, relative to neurons with lower axon elongation. In particular, we set a default distance that axons travel per time step, and introduced a minimum distance that the slowest neurons were limited to. In between these two extremes, we varied the distance that an axon travelled per time step according to the neuron density of its area, changing the axon elongation of infragranular and supragranular neurons in a complementary way. Specifically, the default value of axon elongation was assigned to infragranular compartment neurons in the areas with the lowest neuron density as well as to the supragranular compartment neurons in the areas with the highest neuron density, while the minimum value of axon elongation was assigned to supragranular compartment neurons in the areas with the lowest neuron density as well as the infragranular compartment neurons in the areas with the highest neuron density. As the parameter value for the minimum travelled distance decreased, the divergence between the neurons with shortest and longest axon elongation increased. At baseline, the minimum axon elongation was equal to the default axon elongation, and hence axons elongation was equal for all infra- and supragranular neurons and constant across all source area densities. Independent of whether the elongation of axons within a given time period is actually a relevant factor *in vivo*, this manipulation represents one of many possible ways to implement, *in silico*, differences in cell-intrinsic properties that covary with architectonic differentiation and that account for the fact that infragranular and supragranular compartments can contain neurons with markedly different characteristics [83,112].

We implemented each of the four features at a range of parameter values to systematically evaluate the sensitivity of the outcome measures of interest to variation in the respective property of the *in silico* model. 50 instances of each model implementation were simulated. Since we considered the baseline setting, seven parameter values for each of the two temporal delays, four parameter values for the scaling of supragranular compartment density and six parameter values for the differences in cell-intrinsic properties, we simulated a total of 1250 instances to probe the features individually. In addition, we simulated at least 20 instances each to probe (at a reduced range of parameter values) all pair-wise feature combinations as well as the simultaneous implementation of all four features.

### Analyses

For each simulation instance, we evaluated the resulting connectivity. As mentioned above, we assessed the composition of projections between areas with respect to the distribution of projection origins across laminar compartments, computing the fraction of neurons constituting a projection that originated in the supragranular compartment, *N*_SG_%. The main observation from empirical studies that we set out to replicate was a positive correlation between this supragranular contribution and the relative differentiation of connected areas [1,13,14]. Therefore, we correlated the *N*_SG_% values and neuron density differences, computed as density_source area_—density_target area_, between connected areas that were obtained from the *in silico* model instances, computing Spearman rank correlation coefficients ρ. Since *N*_SG_% is a fraction, its value is quite volatile for very weak projections. As previously done in empirical studies, we therefore applied a threshold to projections strengths prior to computing *N*_SG_% ([8]: at least 20 neurons; [14]: at least 20 neurons, at least 10 neurons in analyses controlling for the conservatism of the applied threshold). We only included projections with a minimum of 10 constituting axons in the analyses presented here. In the simulation, we modelled fewer neurons than are present in an actual cortex, so choosing the less conservative threshold seemed reasonable. The thresholding of connections for the number of constituting axons has been applied to make the percentage of supragranular contribution a more reliable measure. Our simulation does not exactly mirror the primate cortex so faithfully that it would be useful to keep to the same threshold as a matter of principle. Rather, the threshold needs to be so high as to exclude connections with clearly spurious percentages, but not so high that it would exclude too many connections or exclude comparatively weak connections that nonetheless exhibit a systematic bias in supragranular contribution.

To determine whether the correlation coefficient was consistently significant across the distribution resulting from all 50 instances of a model implementation, we computed a left-tailed sign test. Specifically, we tested whether the group of 50 p-values obtained from the rank correlations for each instance had a median value smaller than a significance threshold, α_Spearman_ = 0.05. We considered the sign test significant below α_sign_ = 0.05, and in these cases rejected the null-hypothesis that the median of the group of p-values was not smaller than α_Spearman_.

Previously, we reported that our *in silico* model is capable of generating inter-areal connectivity that is similar to empirically observed cortico-cortical connectivity in the mammalian brain [22]. The two most comprehensive measures that we reported were the correlation between area neuron density and the number of connections an area maintains (area degree) and the classification performance that a classifier which was trained on simulated networks reached when it was applied to empirical data. We wanted to monitor whether the features we introduced in our extended *in silico* model changed the simulated networks with respect to these overarching properties that concern the existence of connections. Therefore, we also report the two measures correlation of neuron density with area degree and simulation-to-empirical classification performance for all implementations of the *in silico* model. Their computation is described in detail in Beul et al. [22]. Briefly, for the correlation between neuron density and area degree we report Spearman rank correlation coefficients ρ and tested the distribution of correlation coefficients across instances of a given model implementation for significance as reported above for the correlation between *N*_SG_% and neuron density differences. To assess simulation-to-empirical classification performance for each simulation instance, we first trained a linear support vector machine to classify projection existence (absent or present) from the z-scores of simulated relative architectonic differentiation (i.e., absolute difference in neuron density) and spatial proximity (i.e., distance) of area pairs. In a second step we used this classifier to classify projection existence in two empirical data sets, from the cat [113] and the macaque [114] cortex. To quantify classification performance, we report the Youden index *J* [115,116], a comprehensive summary measure which takes into account both sensitivity (true positive rate) and specificity (true negative rate), and measures how well a binary classifier operates above chance level with *J* = 0 indicating chance performance and *J* = 1 indicating perfect classification. We considered values of *J* above 0.40 to indicate moderate classification performance and values of above 0.50, where classification performance reaches medium performance between chance level and perfect classification, to indicate good performance (cf. [22,116]). In a third step, we validated the Youden index, assessing how it compared against chance performance by performing permutation analyses. We generated chance performance by classifying 100 times from randomly permuted, non-sensical labels for each simulation instance. We then used a z-test to quantify the probability that the actually observed Youden index value was an element of the distribution of Youden index values obtained at chance performance. See Beul et al. ([22], their Fig 3) for a detailed description of the procedure. Similar to the other measures of interest, we show the distribution of resulting values of *J* across all 50 instances of a model implementation.

All related data can be found in S1 File.

## Supporting information

S1 Fig Supragranular contribution across source area densities. The box plots show the distribution of supragranular contribution (*N*_SG_% values) across density difference values (ranked, see color scale) categorized according to the neuron densities of the source areas (also ranked). For each of the four features (A: delay infragranular compartment, B: delay supragranular compartment, C: supragranular compartment neuron density scaling, D: axon elongation), projections are shown for each of the implemented parameter values. Thus, one row of box plots corresponds to one box in Fig 3. Box plots show distribution across 50 simulation instances per implementation (projections for all 50 instances are collapsed), indicating median (target), interquartile range (box), data range (whiskers) and outliers (circles, outside of 2.7 standard deviations). Parameter values that correspond to baseline (i.e., with no feature implemented), are highlighted in purple.
(PDF)

S2 Fig Correlation of area degree with neuron density. Spearman rank correlation coefficients for the correlation between area degree (number of connections) and area neuron density. (A) delay infragranular compartment, (B) delay supragranular compartment, (C) supragranular compartment neuron density scaling, (D) axon elongation. We used a sign test to determine whether the distribution of associated Spearman rank correlation p-values had a median value smaller than α = 0.05. The result of the sign test is indicated on top; black star: median p < 0.05, red circle: median p ≥ 0.05. Box plots show distribution across 50 simulation instances per implementation, indicating median (line), interquartile range (dark grey box), data range (light grey box) and outliers (circles, outside of 2.7 standard deviations). Parameter values that correspond to baseline (i.e., with no feature implemented), are highlighted in purple.
(PDF)

S3 Fig Simulation-to-empirical classification performance. We trained a classifier on simulated data and used it to classify connection existence from relative differentiation and spatial proximity in the macaque (blue) and cat (green) cortex. Classification performance is indicated by the Youden index *J* for the four implemented features (A: delay infragranular compartment, B: delay supragranular compartment, C: supragranular compartment neuron density scaling, D: axon elongation). Whether the classifier performed better than chance was assessed by a permutation test, where *J* was calculated for prediction from randomly permuted labels and a z-test was performed. We used a sign test to determine whether the distribution of associated z-test p-values had a median value smaller than α = 0.05. The result of the sign test is indicated on top; black star: performance better than chance with median p < 0.05, red circle: performance not better than chance with median p ≥ 0.05. Box plots show distribution across 50 simulation instances per implementation, indicating median (line), interquartile range (dark grey box), data range (light grey box) and outliers (circles, outside of 2.7 standard deviations). Parameter values that correspond to baseline (i.e., with no feature implemented), are highlighted in purple.
(PDF)

S4 Fig Correlation between neuron density difference and ratio of supragranular neurons to total neurons. (A) The correlation between neuron density difference and the ratio of supragranular neurons to total neurons (supra-to-total ratio) is depicted in relation to the individual correlation between supragranular contribution and the supra-to-total ratio (which is the same correlation as depicted on the abscissa in Fig 4A). (B) The partial correlation between neuron density difference and supra-to-total ratio, controlled for supragranular contribution, is depicted in relation to the partial correlation between supragranular contribution and the supra-to-total ratio, controlled for neuron density difference (which is the same correlation as depicted on the abscissa in Fig 4B). The correlation between density difference and supra-to-total ratio weakens somewhat if controlled for supragranular contribution, while the correlation between supragranular contribution and supra-to-total ratio is hardly affected by controlling for the difference in neuron density between connected areas. (C) The data underlying the reported correlations between density difference and supra-to-total ratio are shown for each parameter value of the supragranular neuron density scaling parameter (1 to 5). The distribution for the parameter value of 1 (which is the baseline setting) is flat at 0.5, because for all areas the supragranular neuron density was equal to the infragranular neuron density. This also means that no correlation can be computed for this parameter value. Therefore, it does not appear in (A) or (B). Since we report Spearman rank correlations between density difference and neuron ratio, the correlation coefficient ρ is equal across all values of the supragranular density scaling parameter in (A). If an individual Pearson correlation is computed instead, there is a slight spread of the correlation coefficient r across parameter values (2: r = 0.71, 3: r = 0.70, 4: r = 0.69, 5: r = 0.67).
(PDF)

S5 Fig Axon elongation without ordered succession of neuron density values. Results for implementation of scaling in axon elongation with randomly assigned area neuron densities. That is, this implementation lacked the ordered gradient of areas with higher neuron density forming at later points in time that was present in the other implementations. (A) Spearman rank correlation coefficients for the correlation between area degree (number of connections) and area neuron density, as in S2 Fig. (B) Classification performance for simulation-to-empirical classification performance from relative differentiation and spatial proximity, as in S3 Fig. The results of sign tests are indicated on top; black star: performance better than chance with median p < 0.05, red circle: performance not better than chance with median p ≥ 0.05. Box plots show distribution across 50 simulation instances per implementation, indicating median (line), interquartile range (dark grey box), data range (light grey box) and outliers (circles, outside of 2.7 standard deviations).
(PDF)

S6 Fig Pairwise combination of features. Spearman rank correlation coefficients for the correlation between the supragranular contribution of a projection and the neuron density difference between the connected areas. We simulated implementations of all pairwise combinations of features at a reduced set of parameter values. (A) delay infragranular compartment and delay supragranular compartment, (B) supragranular compartment neuron density scaling and axon elongation, (C) delay infragranular compartment and supragranular compartment neuron density scaling, (D) delay supragranular compartment and supragranular compartment neuron density scaling, (E) delay infragranular compartment and axon elongation, (F) delay supragranular compartment and axon elongation.
(PDF)

S7 Fig Combination of all features. Spearman rank correlation coefficients for the correlation between the supragranular contribution of a projection and the neuron density difference between the connected areas. We simulated implementations of all four features simultaneously, at a reduced set of parameter values. (A) Correlation coefficients for the correlation of supragranular contribution values with neuron density difference between connected areas. (B) Partial correlation coefficients for the correlation of supragranular contribution value with neuron density difference, controlling for the supra-to-total neuron ratio (as in Fig 4B).
(PDF)

S1 File Data underlying the presented findings. (XLSX)
