# Supplementary material for: Systematic modelling of the development of laminar projection origins in the cerebral cortex: Interactions of spatio-temporal patterns of neurogenesis and cellular heterogeneity
Source: PLoS Comput Biol. 2020 Oct 13;16(10):e1007991. doi: 10.1371/journal.pcbi.1007991 (PMC7553356; doi:10.1371/journal.pcbi.1007991)
Supplement: S1 Fig — The box plots show the distribution of supragranular contribution (NSG% values) across density difference values (ranked, see color scale) categorized according to the neuron densities of the source areas (also ranked). For each of the four features (A: delay infragranular compartment, B: delay supragranular compartment, C: supragranular compartment neuron density scaling, D: axon elongation), projections are shown for each of the implemented parameter values. Thus, one row of box plots corresponds to one box in Fig 3. Box plots show distribution across 50 simulation instances per implementation (projections for all 50 instances are collapsed), indicating median (target), interquartile range (box), data range (whiskers) and outliers (circles, outside of 2.7 standard deviations). Parameter values that correspond to baseline (i.e., with no feature implemented), are highlighted in purple. (PDF) [file pcbi.1007991.s001.pdf]

Supplementary Figure S1

A delay infragranular compartment growth

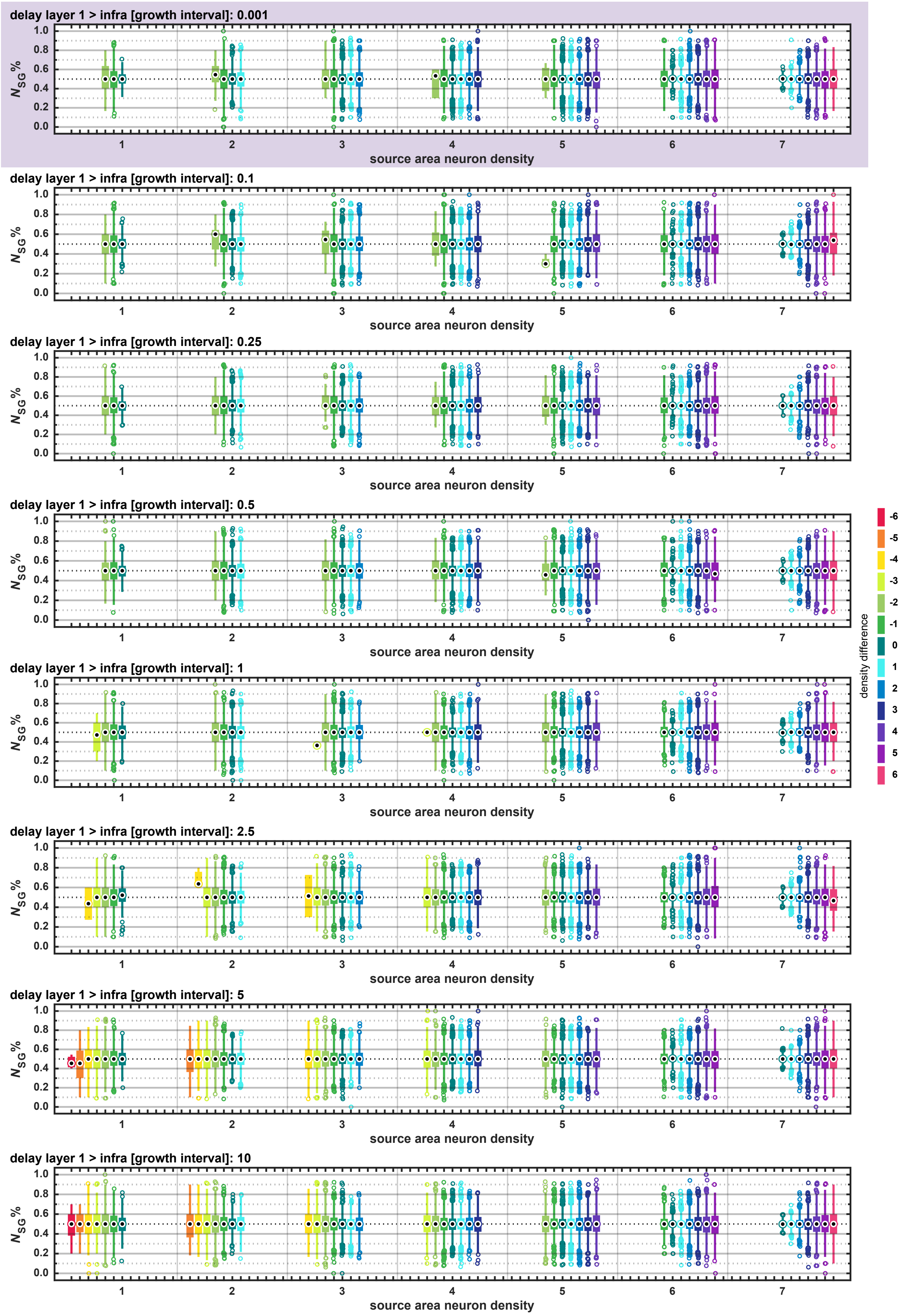

Supplementary Figure S1

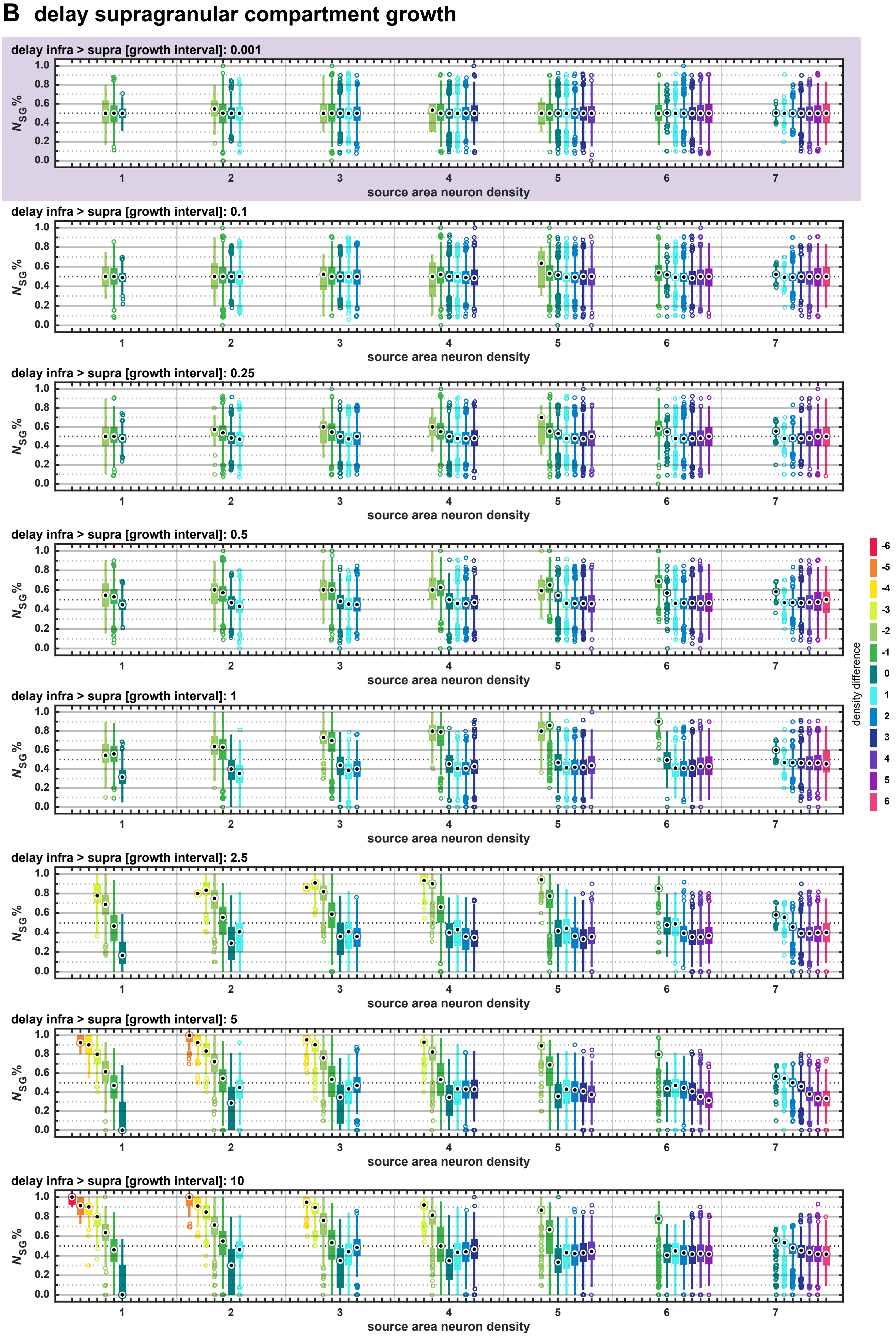

Supplementary Figure S1

C supragranular compartment neuron density

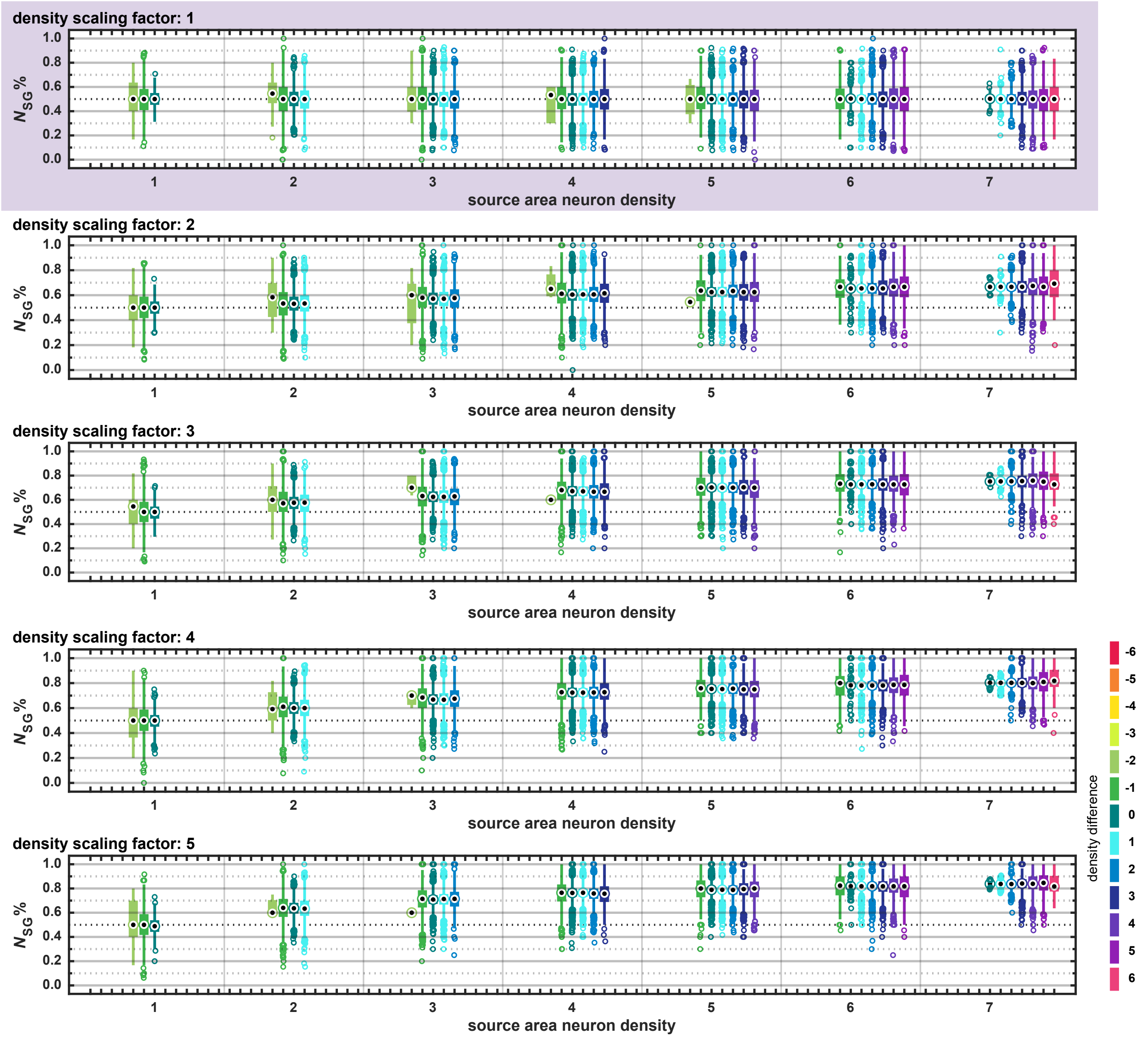

Supplementary Figure S1

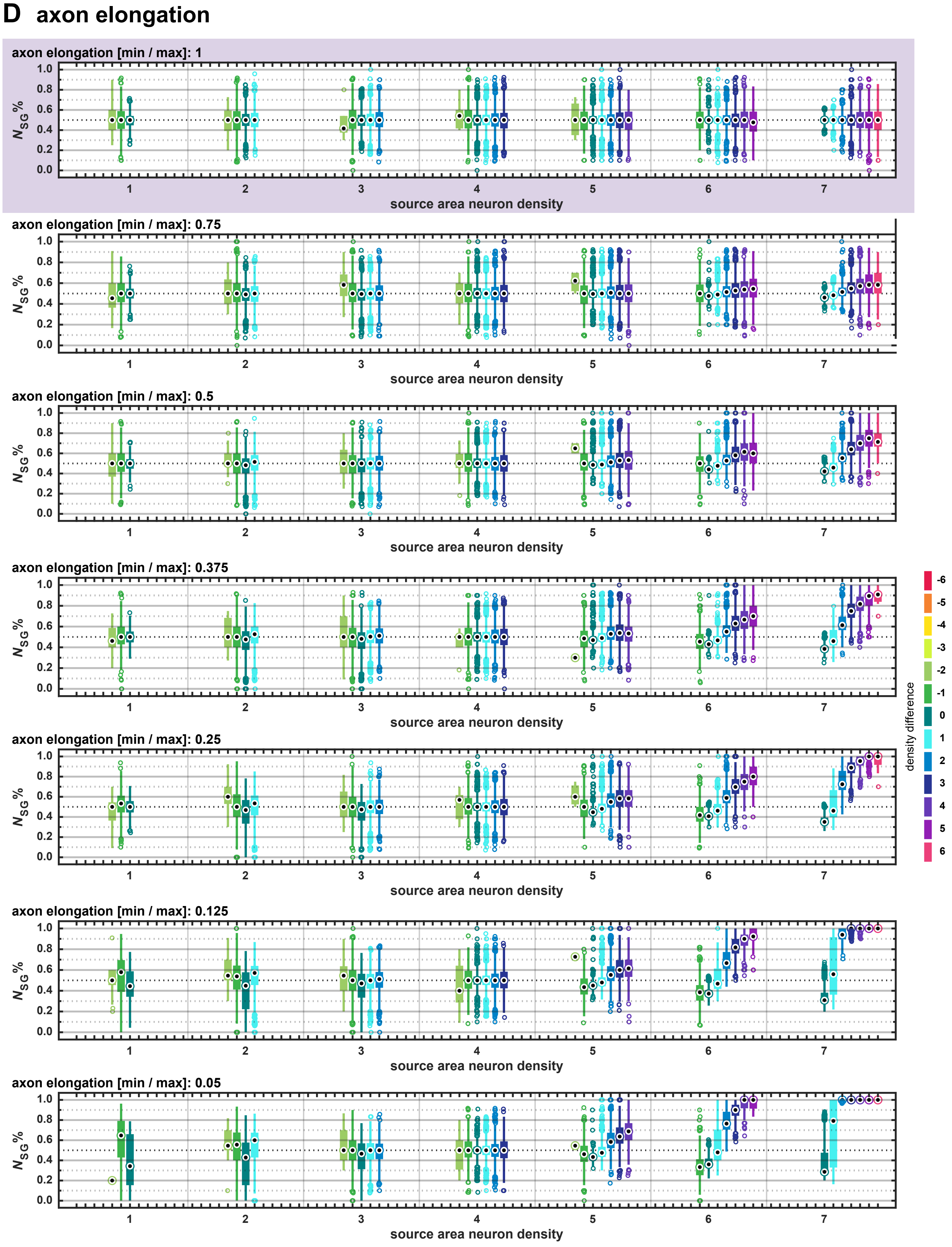

SUPPLEMENTARY FIGURE S1: SUPRAGRANULAR CONTRIBUTION ACROSS SOURCE AREA DENSITIES.

The box plots show the distribution of supragranular contribution (N_{SG}% values) across density difference values (ranked, see color scale) categorized according to the neuron densities of the source areas (also ranked). For each of the four features (A: delay infragranular compartment, B: delay supragranular compartment, C: supragranular compartment neuron density scaling, D: axon elongation), projections are shown for each of the implemented parameter values. Thus, one row of box plots corresponds to one box in Figure 3. Box plots show distribution across 50 simulation instances per implementation (projections for all 50 instances are collapsed), indicating median (target), interquartile range (box), data range (whiskers) and outliers (circles, outside of 2.7 standard deviations). Parameter values that correspond to baseline (i.e., with no feature implemented), are highlighted in purple.
