# Supplementary material for: Systematic modelling of the development of laminar projection origins in the cerebral cortex: Interactions of spatio-temporal patterns of neurogenesis and cellular heterogeneity
Source: PLoS Comput Biol. 2020 Oct 13;16(10):e1007991. doi: 10.1371/journal.pcbi.1007991 (PMC7553356; doi:10.1371/journal.pcbi.1007991)
Supplement: S2 Fig — Spearman rank correlation coefficients for the correlation between area degree (number of connections) and area neuron density. (A) delay infragranular compartment, (B) delay supragranular compartment, (C) supragranular compartment neuron density scaling, (D) axon elongation. We used a sign test to determine whether the distribution of associated Spearman rank correlation p-values had a median value smaller than α = 0.05. The result of the sign test is indicated on top; black star: median p < 0.05, red circle: median p ≥ 0.05. Box plots show distribution across 50 simulation instances per implementation, indicating median (line), interquartile range (dark grey box), data range (light grey box) and outliers (circles, outside of 2.7 standard deviations). Parameter values that correspond to baseline (i.e., with no feature implemented), are highlighted in purple. (PDF) [file pcbi.1007991.s002.pdf]

Supplementary Figure S2

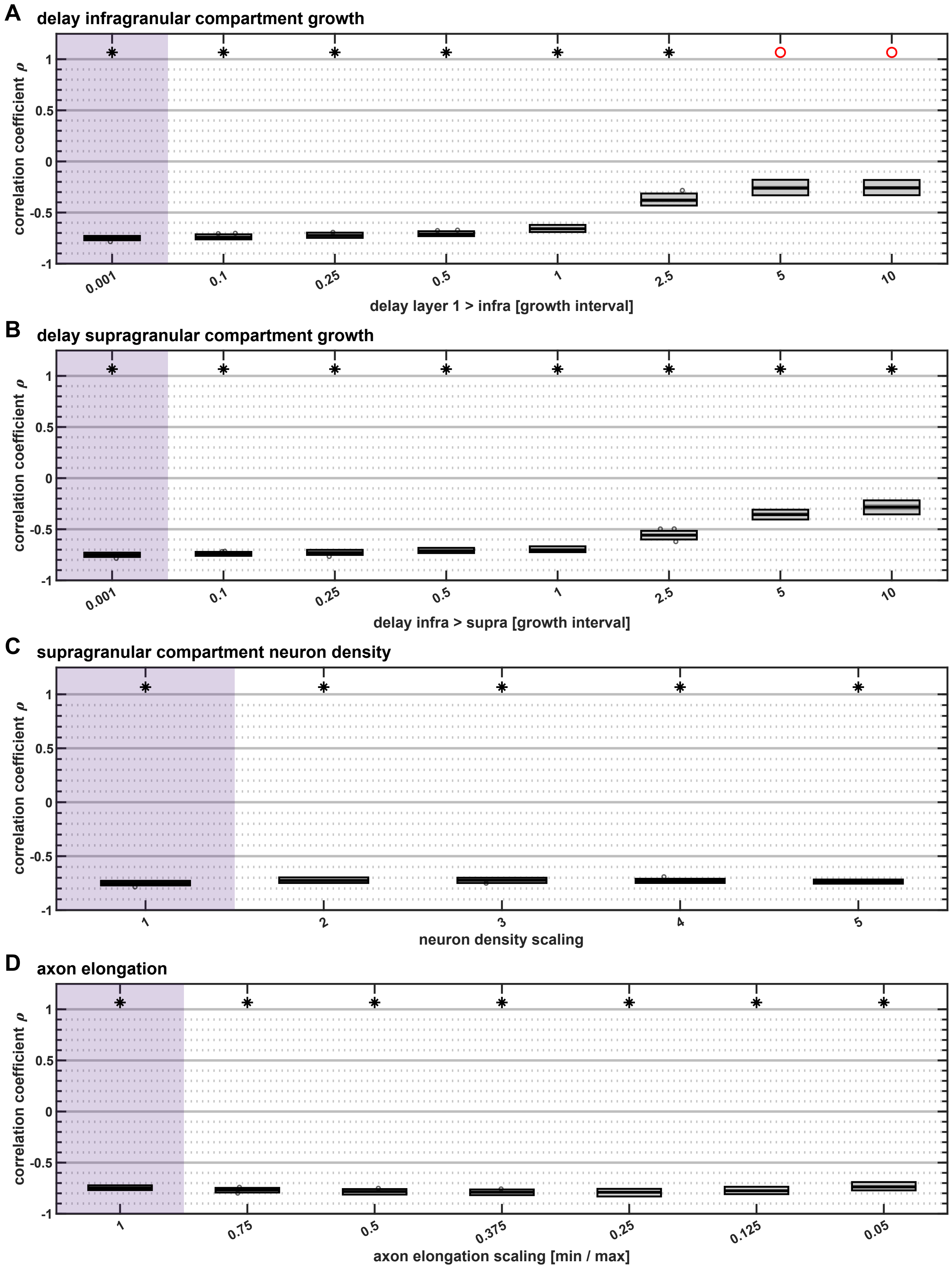

SUPPLEMENTARY FIGURE S2: CORRELATION OF AREA DEGREE WITH NEURON DENSITY.

Spearman rank correlation coefficients for the correlation between area degree (number of connections) and area neuron density. We used a sign test to determine whether the distribution of associated Spearman rank correlation p-values had a median value smaller than $\alpha = 0.05$. The result of the sign test is indicated on top; black star: median $p < 0.05$, red circle: median $p \geq 0.05$. Box plots show distribution across 50 simulation instances per implementation, indicating median (line), interquartile range (dark grey box), data range (light grey box) and outliers (circles, outside of 2.7 standard deviations). Parameter values that correspond to baseline (i.e., with no feature implemented), are highlighted in purple.
