# Supplementary material for: Systematic modelling of the development of laminar projection origins in the cerebral cortex: Interactions of spatio-temporal patterns of neurogenesis and cellular heterogeneity
Source: PLoS Comput Biol. 2020 Oct 13;16(10):e1007991. doi: 10.1371/journal.pcbi.1007991 (PMC7553356; doi:10.1371/journal.pcbi.1007991)
Supplement: S3 Fig — We trained a classifier on simulated data and used it to classify connection existence from relative differentiation and spatial proximity in the macaque (blue) and cat (green) cortex. Classification performance is indicated by the Youden index J for the four implemented features (A: delay infragranular compartment, B: delay supragranular compartment, C: supragranular compartment neuron density scaling, D: axon elongation). Whether the classifier performed better than chance was assessed by a permutation test, where J was calculated for prediction from randomly permuted labels and a z-test was performed. We used a sign test to determine whether the distribution of associated z-test p-values had a median value smaller than α = 0.05. The result of the sign test is indicated on top; black star: performance better than chance with median p < 0.05, red circle: performance not better than chance with median p ≥ 0.05. Box plots show distribution across 50 simulation instances per implementation, indicating median (line), interquartile range (dark grey box), data range (light grey box) and outliers (circles, outside of 2.7 standard deviations). Parameter values that correspond to baseline (i.e., with no feature implemented), are highlighted in purple. (PDF) [file pcbi.1007991.s003.pdf]

Supplementary Figure S3

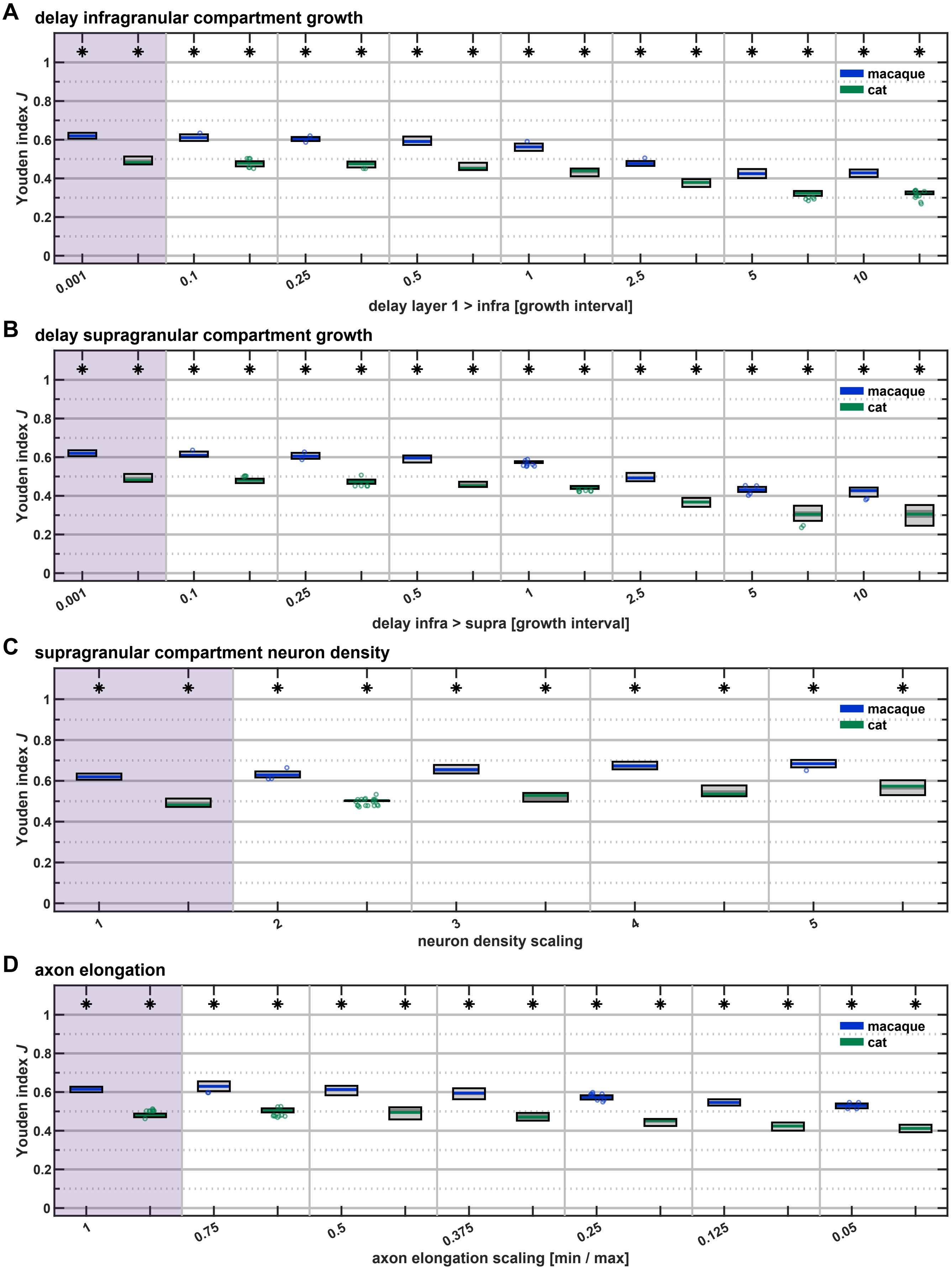

SUPPLEMENTARY FIGURE S3: SIMULATION-TO-EMPIRICAL CLASSIFICATION PERFORMANCE.

We trained a classifier on simulated data and used it to classify connection existence from relative differentiation and spatial proximity in the macaque (blue) and cat (green) cortex. Classification performance is indicated by the Youden index *J* for the four implemented features. Whether the classifier performed better than chance was assessed by a permutation test, where *J* was calculated for prediction from randomly permuted labels and a z-test was performed. We used a sign test to determine whether the distribution of associated z-test p-values had a median value smaller than $\alpha = 0.05$. The result of the sign test is indicated on top; black star: performance better than chance with median $p < 0.05$, red circle: performance not better than chance with median $p \geq 0.05$. Box plots show distribution across 50 simulation instances per implementation, indicating median (line), interquartile range (dark grey box), data range (light grey box) and outliers (circles, outside of 2.7 standard deviations). Parameter values that correspond to baseline (i.e., with no feature implemented), are highlighted in purple.
