# Supplementary material for: Systematic modelling of the development of laminar projection origins in the cerebral cortex: Interactions of spatio-temporal patterns of neurogenesis and cellular heterogeneity
Source: PLoS Comput Biol. 2020 Oct 13;16(10):e1007991. doi: 10.1371/journal.pcbi.1007991 (PMC7553356; doi:10.1371/journal.pcbi.1007991)
Supplement: S4 Fig — (A) The correlation between neuron density difference and the ratio of supragranular neurons to total neurons (supra-to-total ratio) is depicted in relation to the individual correlation between supragranular contribution and the supra-to-total ratio (which is the same correlation as depicted on the abscissa in Fig 4A). (B) The partial correlation between neuron density difference and supra-to-total ratio, controlled for supragranular contribution, is depicted in relation to the partial correlation between supragranular contribution and the supra-to-total ratio, controlled for neuron density difference (which is the same correlation as depicted on the abscissa in Fig 4B). The correlation between density difference and supra-to-total ratio weakens somewhat if controlled for supragranular contribution, while the correlation between supragranular contribution and supra-to-total ratio is hardly affected by controlling for the difference in neuron density between connected areas. (C) The data underlying the reported correlations between density difference and supra-to-total ratio are shown for each parameter value of the supragranular neuron density scaling parameter (1 to 5). The distribution for the parameter value of 1 (which is the baseline setting) is flat at 0.5, because for all areas the supragranular neuron density was equal to the infragranular neuron density. This also means that no correlation can be computed for this parameter value. Therefore, it does not appear in (A) or (B). Since we report Spearman rank correlations between density difference and neuron ratio, the correlation coefficient ρ is equal across all values of the supragranular density scaling parameter in (A). If an individual Pearson correlation is computed instead, there is a slight spread of the correlation coefficient r across parameter values (2: r = 0.71, 3: r = 0.70, 4: r = 0.69, 5: r = 0.67). (PDF) [file pcbi.1007991.s004.pdf]

Supplementary Figure S4

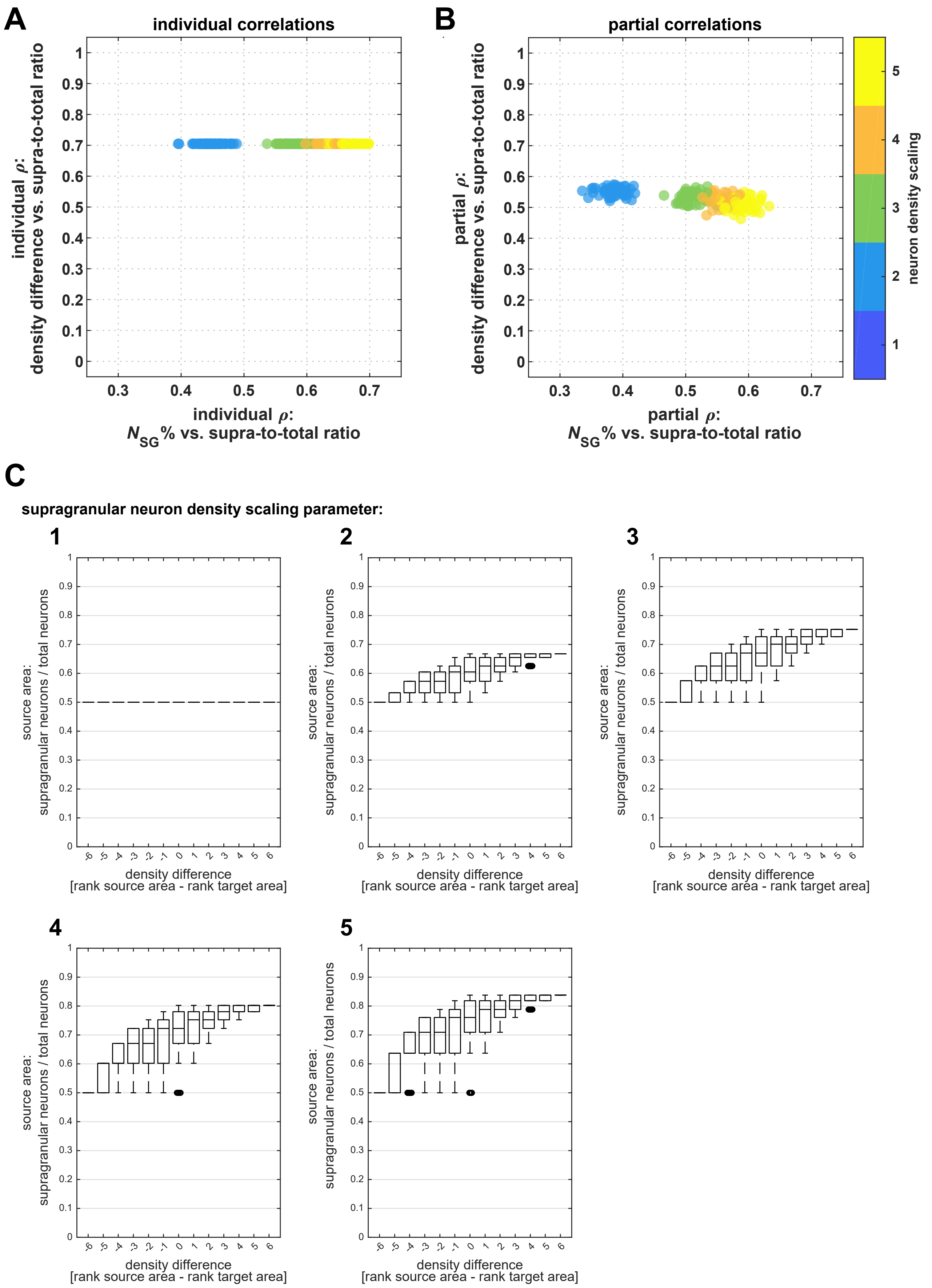

SUPPLEMENTARY FIGURE S4: CORRELATION BETWEEN NEURON DENSITY DIFFERENCE AND RATIO OF SUPRAGRANULAR NEURONS TO TOTAL NEURONS.

(A) The correlation between neuron density difference and the ratio of supragranular neurons to total neurons (supra-to-total ratio) is depicted in relation to the individual correlation between supragranular contribution and the supra-to-total ratio (which is the same correlation as depicted on the abscissa in Figure 4A). (B) The partial correlation between neuron density difference and supra-to-total ratio, controlled for supragranular contribution, is depicted in relation to the partial correlation between supragranular contribution and the supra-to-total ratio, controlled for neuron density difference (which is the same correlation as depicted on the abscissa in Figure 4B). The correlation between density difference and supra-to-total ratio weakens somewhat if controlled for supragranular contribution, while the correlation between supragranular contribution and supra-to-total ratio is hardly affected by controlling for the difference in neuron density between connected areas. (C) The data underlying the reported correlations between density difference and supra-to-total ratio are shown for each parameter value of the supragranular neuron density scaling parameter (1 to 5). The distribution for the parameter value of 1 (which is the baseline setting) is flat at 0.5, because for all areas the supragranular neuron density was equal to the infragranular neuron density. This also means that no correlation can be computed for this parameter value. Therefore, it does not appear in (A) or (B). Since we report Spearman rank correlations between density difference and neuron ratio, the correlation coefficient ρ is equal across all values of the supragranular density scaling parameter in (A). If an individual Pearson correlation is computed instead, there is a slight spread of the correlation coefficient r across parameter values (2: $r = 0.71$, 3: $r = 0.70$, 4: $r = 0.69$, 5: $r = 0.67$).
