# Supplementary material for: Systematic modelling of the development of laminar projection origins in the cerebral cortex: Interactions of spatio-temporal patterns of neurogenesis and cellular heterogeneity
Source: PLoS Comput Biol. 2020 Oct 13;16(10):e1007991. doi: 10.1371/journal.pcbi.1007991 (PMC7553356; doi:10.1371/journal.pcbi.1007991)
Supplement: S5 Fig — Results for implementation of scaling in axon elongation with randomly assigned area neuron densities. That is, this implementation lacked the ordered gradient of areas with higher neuron density forming at later points in time that was present in the other implementations. (A) Spearman rank correlation coefficients for the correlation between area degree (number of connections) and area neuron density, as in S2 Fig. (B) Classification performance for simulation-to-empirical classification performance from relative differentiation and spatial proximity, as in S3 Fig. The results of sign tests are indicated on top; black star: performance better than chance with median p < 0.05, red circle: performance not better than chance with median p ≥ 0.05. Box plots show distribution across 50 simulation instances per implementation, indicating median (line), interquartile range (dark grey box), data range (light grey box) and outliers (circles, outside of 2.7 standard deviations). (PDF) [file pcbi.1007991.s005.pdf]

Supplementary Figure S5

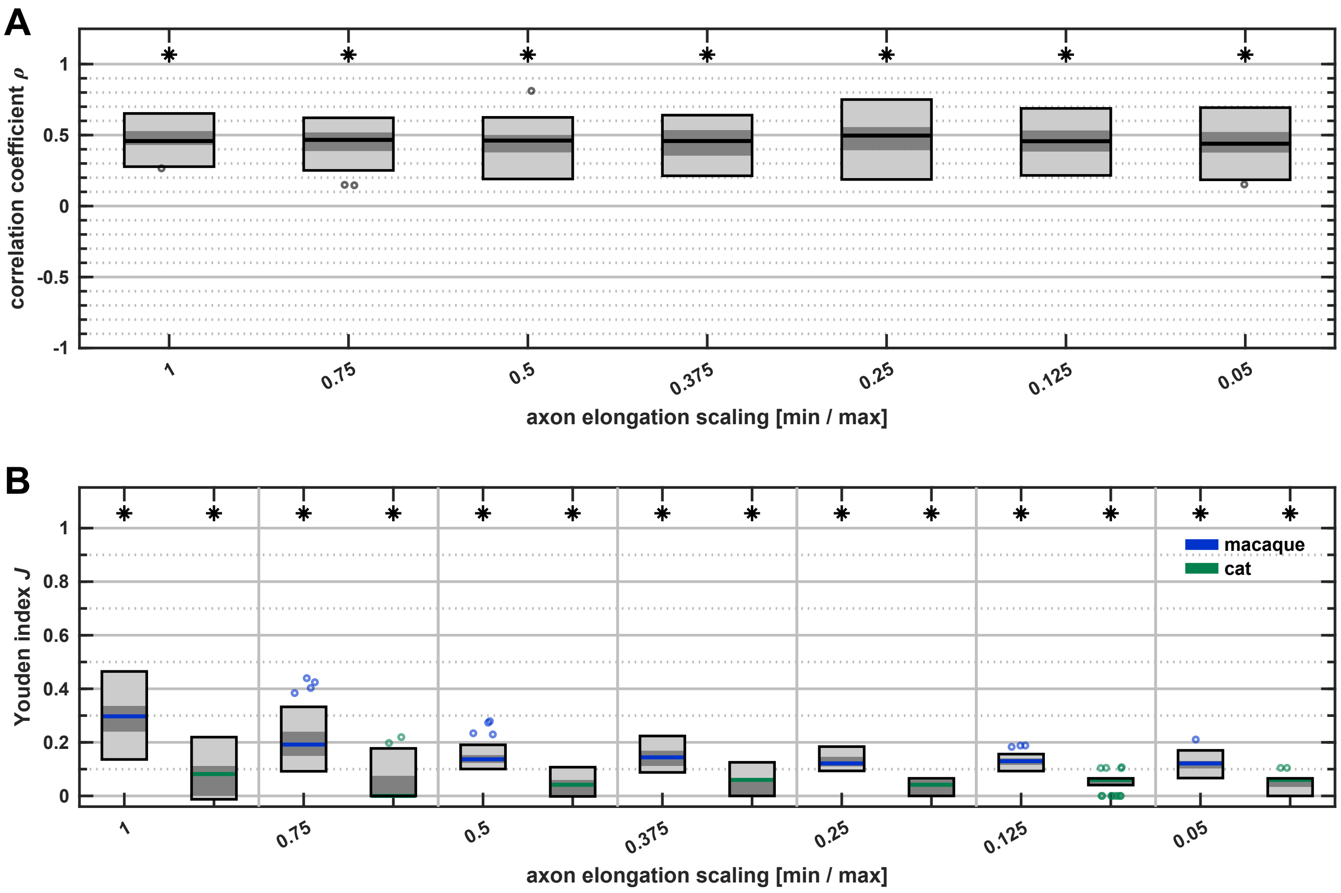

SUPPLEMENTARY FIGURE S5: AXON ELONGATION WITHOUT ORDERED SUCCESSION OF NEURON DENSITY VALUES.

Results for implementation of scaling in axon elongation with randomly assigned area neuron densities. That is, this implementation lacked the ordered gradient of areas with higher neuron density forming at later points in time that was present in the other implementations. (A) Spearman rank correlation coefficients for the correlation between area degree (number of connections) and area neuron density, as in Supplementary Figure S2. (B) Classification performance for simulation-to-empirical classification performance from relative differentiation and spatial proximity, as in Supplementary Figure S3. The results of sign tests are indicated on top; black star: performance better than chance with median $p < 0.05$, red circle: performance not better than chance with median $p \geq 0.05$. Box plots show distribution across 50 simulation instances per implementation, indicating median (line), interquartile range (dark grey box), data range (light grey box) and outliers (circles, outside of 2.7 standard deviations).
