# Supplementary material for: Systematic modelling of the development of laminar projection origins in the cerebral cortex: Interactions of spatio-temporal patterns of neurogenesis and cellular heterogeneity
Source: PLoS Comput Biol. 2020 Oct 13;16(10):e1007991. doi: 10.1371/journal.pcbi.1007991 (PMC7553356; doi:10.1371/journal.pcbi.1007991)
Supplement: S6 Fig — Spearman rank correlation coefficients for the correlation between the supragranular contribution of a projection and the neuron density difference between the connected areas. We simulated implementations of all pairwise combinations of features at a reduced set of parameter values. (A) delay infragranular compartment and delay supragranular compartment, (B) supragranular compartment neuron density scaling and axon elongation, (C) delay infragranular compartment and supragranular compartment neuron density scaling, (D) delay supragranular compartment and supragranular compartment neuron density scaling, (E) delay infragranular compartment and axon elongation, (F) delay supragranular compartment and axon elongation. (PDF) [file pcbi.1007991.s006.pdf]

Supplementary Figure S6

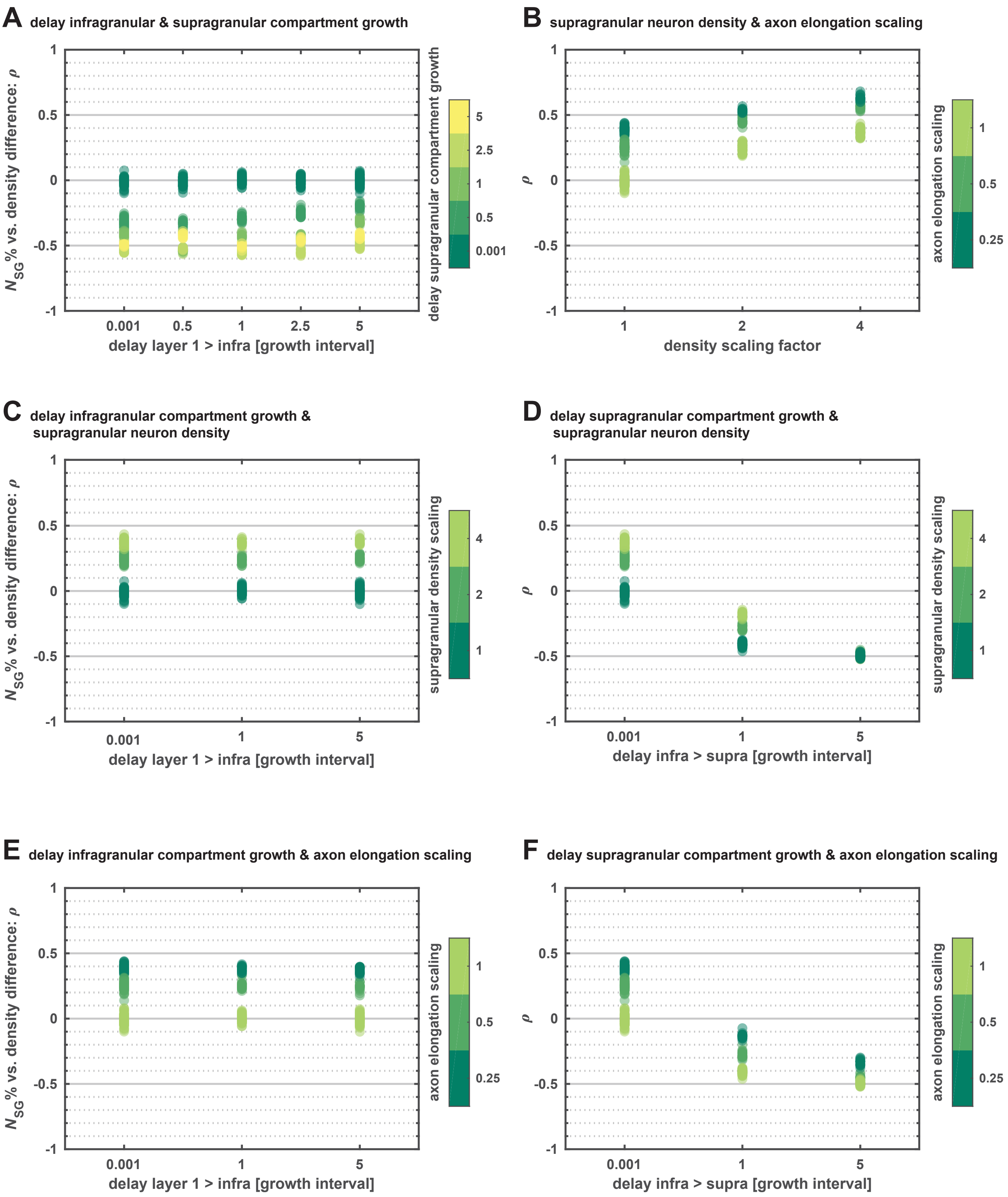

Supplementary Figure S6: Pairwise combination of features.

Spearman rank correlation coefficients for the correlation between the supragranular contribution of a projection and the neuron density difference between the connected areas. We simulated implementations of all pairwise combinations of features at a reduced set of parameter values.
