# Supplementary material for: Systematic modelling of the development of laminar projection origins in the cerebral cortex: Interactions of spatio-temporal patterns of neurogenesis and cellular heterogeneity
Source: PLoS Comput Biol. 2020 Oct 13;16(10):e1007991. doi: 10.1371/journal.pcbi.1007991 (PMC7553356; doi:10.1371/journal.pcbi.1007991)
Supplement: S7 Fig — Spearman rank correlation coefficients for the correlation between the supragranular contribution of a projection and the neuron density difference between the connected areas. We simulated implementations of all four features simultaneously, at a reduced set of parameter values. (A) Correlation coefficients for the correlation of supragranular contribution values with neuron density difference between connected areas. (B) Partial correlation coefficients for the correlation of supragranular contribution value with neuron density difference, controlling for the supra-to-total neuron ratio (as in Fig 4B). (PDF) [file pcbi.1007991.s007.pdf]

Supplementary Figure S7

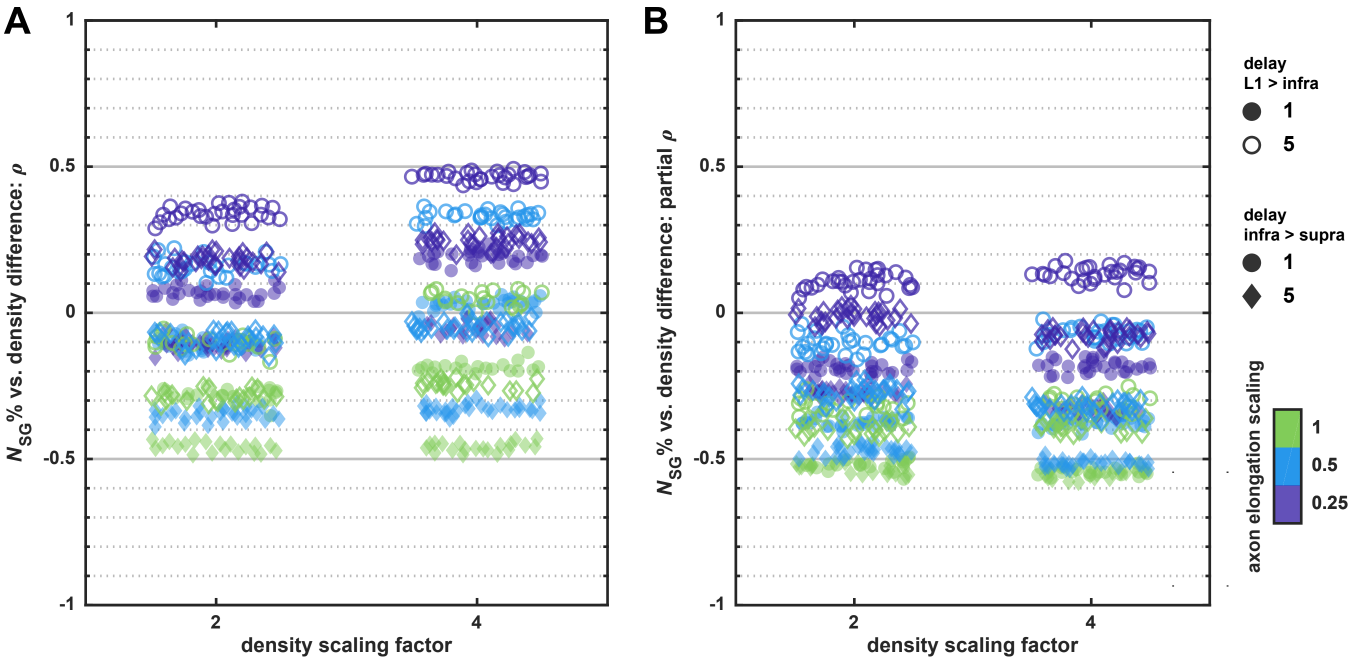

Supplementary Figure S7: Combination of all features.

Spearman rank correlation coefficients for the correlation between the supragranular contribution of a projection and the neuron density difference between the connected areas. We simulated implementations of all four features simultaneously, at a reduced set of parameter values. (A) Correlation coefficients for the correlation of supragranular contribution values with neuron density difference between connected areas. (B) Partial correlation coefficients for the correlation of supragranular contribution value with neuron density difference, controlling for the supra-to-total neuron ratio (as in Figure 4B).
